# Integrating water exclusion theory into *β*contacts to predict binding free energy changes and binding hot spots

**DOI:** 10.1186/1471-2105-15-57

**Published:** 2014-02-26

**Authors:** Qian Liu, Steven CH Hoi, Chee Keong Kwoh, Limsoon Wong, Jinyan Li

**Affiliations:** 1 Advanced Analytics Institute and Center for Health Technologies, Faculty of Engineering and IT, University of Technology, Sydney, Australia; 2 School of Computer Engineering, Nanyang Technological University, Singapore 639798, Singapore; 3 School of Computing, National University of Singapore, Singapore 117417, Singapore

## Abstract

**Background:**

Binding free energy and binding hot spots at protein-protein interfaces are two important research areas for understanding protein interactions. Computational methods have been developed previously for accurate prediction of binding free energy change upon mutation for interfacial residues. However, a large number of interrupted and unimportant atomic contacts are used in the training phase which caused accuracy loss.

**Results:**

This work proposes a new method, *βACV*_*ASA*_, to predict the change of binding free energy after alanine mutations. *βACV*_*ASA*_ integrates accessible surface area (ASA) and our newly defined *β* contacts together into an atomic contact vector (ACV). A *β* contact between two atoms is a direct contact without being interrupted by any other atom between them. A *β* contact’s potential contribution to protein binding is also supposed to be inversely proportional to its ASA to follow the water exclusion hypothesis of binding hot spots. Tested on a dataset of 396 alanine mutations, our method is found to be superior in classification performance to many other methods, including Robetta, FoldX, HotPOINT, an ACV method of *β* contacts without ASA integration, and ACV_*ASA*_ methods (similar to *βACV*_*ASA*_ but based on distance-cutoff contacts). Based on our data analysis and results, we can draw conclusions that: (i) our method is powerful in the prediction of binding free energy change after alanine mutation; (ii) *β* contacts are better than distance-cutoff contacts for modeling the well-organized protein-binding interfaces; (iii) *β* contacts usually are only a small fraction number of the distance-based contacts; and (iv) water exclusion is a necessary condition for a residue to become a binding hot spot.

**Conclusions:**

*βACV*_*ASA*_ is designed using the advantages of both *β* contacts and water exclusion. It is an excellent tool to predict binding free energy changes and binding hot spots after alanine mutation.

## Background

A binding hot spot is a small area in a protein binding interface whose mutation can lead to a big change in binding free energy. The determination of its accurate location in the interface is a fundamental problem in structural biology, and is useful for applications such as rational drug design and protein engineering [1]. In wet labs, a residue’s contribution to binding free energy can be determined through mutation experiments. For example, alanine scanning mutagenesis [2] mutates interfacial residues individually into an alanine, and then measures the change of binding free energy (*ΔΔG*) to quantify the contribution of the side chain of the mutated residue. Based on these wet-lab experimental outcomes and databases [3-6], it has been reported that binding free energy is unevenly distributed in protein interfaces [7]. In fact, there are always a small fraction of interfacial residues—the binding hot spot—which make major contribution to the binding [7,8] with *ΔΔG*≥2 kcal/mol [3]. But wet-lab experiments are both time and cost expensive. Reliable computational methods are thus needed for accurate prediction of binding free energy change.

FoldX [9,10], Robetta [11,12] and CC/PBSA [13] are some well-known physics-based methods for this prediction problem. These methods use empirical terms (such as hydrogen bonds), the van der Waals terms and Coulomb electrostatics to learn a linear function for estimating the effect on the change of binding free energy after residue mutations. However, the predicted energy by these methods has a large discrepancy from experimentally measured *ΔΔG*[14]. Thus, other methods have been proposed to qualitatively identify binding hot spots. For example, protein sequences are used by [15] and ISIS [16], while protein tertiary structures are used together with docking techniques by [17]. Protein quaternary structures have been also widely used [18]. For example, Hotsprint [19] and HotPOINT [20] generate rules to identify binding hot spots from features such as conservation, accessible surface area (ASA), residue propensity and/or residue pairwise potentials. Machine learning models are also widely used for predicting binding hot spots. Decision trees are used in MINERVA [14] to induce rules at different levels of protein information including structure, sequence and molecular interactions. Later, machine learning algorithms SVM and its ensemble are employed to combine energetic terms such as van der Waals potentials, solvation energy, hydrogen bonds and Coulomb electrostatics, and/or other protein sequences and structure information for a better hot spot prediction performance. Recently, Bayesian Networks are used to combine three main sources of information related to conservation, FoldX-calculated *ΔΔG* and atomic contacts for a novel probabilistic model of binding hot spots prediction [21]. Very recently, random forests have been proposed to predict hot spots [22] by using structural neighborhood properties of mutated residues and other conventional physicochemical features [23,24]. Besides alanine mutations, hot spots after mutations to any other type of residues are also investigated [6] and their binding free energy changes can be predicted [13,25] with good performance. Several of these methods are also assessed in a community-wide test for predicting mutation effects on protein-protein interaction affinity [26].

In spite of intensive research, the prediction still needs a big improvement. The existing methods usually used those atomic contacts based on Voronoi diagram or simply defined by a distance threshold with little consideration on the local atomic organization of the contacts. If the distance threshold is too large, e.g., larger than 6 Å, an atomic contact between two atoms *i* and *j* may have no direct contact area, because the space between *i* and *j* can accommodate other atoms. Such interrupted contacts constitute a large proportion of the traditionally used contacts. It is highly questionable whether they are really important to protein binding. In fact, important contacts in hot spot prediction [10,11] or those closely related to binding hot spots [14] are generally not interrupted, such as hydrogen bonds, salt bridges and *π*−*π* contacts.

To overcome these drawbacks, we propose a novel classifier *β*ACV_*ASA*_ for predicting *ΔΔG* and binding hot spots. The main idea of *β*ACV_*ASA*_ is to use atomic contact vector (ACV) of *β* contacts (that’s why our classifier is named *β*ACV for short) instead of distance-cutoff contacts. *β* contact, found on *β*-skeletons [27], is our newly defined contact [28]. A *β* contact between two atoms restricts that there is no other atoms between these two atoms, and requires that the two atoms should have enough direct contact area to form an interaction. The definition of *β* contacts can filter out a lot of unimportant and interrupted distance-cutoff contacts. Our analysis has found that *β* contacts are only a small fraction number of those contacts based on a distance threshold [28], but they are effective to distinguish crystal packing from homodimers [28] and to predict protein-ligand binding affinity [29].

Another important idea is that the relative ASA properties are integrated by our *β*ACV classifier based on the water exclusion hypothesis of binding hot spots. The water exclusion hypothesis states that the topological shape of a binding hot spot and its surrounding residues can be characterized as an O-ring structure [3]. Few residues on the O-ring, which are largely exposed to bulk solvent water, can contribute significantly to the protein binding. Thus in *β*ACV, the energy contribution of a *β* contact to protein binding is required to be inversely proportional to its ASA.

Our *β*ACV_*ASA*_ was tested on a dataset of 396 alanine mutations to show its superior performance. We compared *β*ACV_*ASA*_ with the following methods: (i) ACV methods using distance-cutoff contacts to reveal the importance of *β* contacts to protein binding; (ii) a *β*ACV method without ASA integration to confirm whether the water exclusion theory is necessary for binding hot spots; and (iii) several widely-used state-of-the-art methods such as Robetta, FoldX, HotPOINT and KFC to show the overall better prediction capability of *β*ACV_*ASA*_.

## Methods

### Dataset

The data stored in the ASEdb database [3] and the mutations in BID [4] having *ΔΔG* measurements are both used for evaluating our method. In total, our dataset contains 22 protein-protein complexes (detailed in Additional file 1: Table S2). All of them have quaternary structures in PDB and meet the following three requirements. First, no redundancy exists among these protein complexes. Given two protein complexes (e.g., interacted pair A and B, and interacted pair C and D), a sequence identity is calculated through BLAST with the default setting for A and C, A and D, B and C, and B and D, denoted by *S*(*A*,*C*), *S*(*A*,*D*), *S*(*B*,*C*) and *S*(*B*,*D*) respectively. These two protein complexes are redundant if *S*(*A*,*C*)≥40*%* and *S*(*B*,*D*)≥40*%*, or *S*(*A*,*D*)≥40*%* and *S*(*B*,*C*)≥40*%*. According to this criterion, most of the protein complexes in our dataset are non-redundant. For those redundant complexes, the mutations in the similar proteins must be in different positions. Our requirement on this sequence identity is reasonable, since atomic contacts and ASA used in this work are derived from complexes only, rather than from sequences (such as required by conservation scores). Secondly, only alanine mutations are considered. Thirdly, mutated atoms before mutation must have at least one distance-cutoff atomic contact with the partner proteins. The mutated atoms are those atoms except N, CA, C, O and CB. Under these requirements, our dataset has 396 alanine mutations (detailed in Additional file 1: Table S3). Of these mutations, 86 are binding hot spot residues having *ΔΔG*≥2 kcal/mol.

### Atomic *β* contacts in protein binding interfaces

Atomic *β* contact is a recently proposed notion of atomic contacts for modeling the well-organized protein 3D structures [28]. Its detail can be found in [28]. For easy reference, we give a brief description of *β* contacts and a simple method to produce *β* contacts from a protein quaternary structure.

#### Atomic *β* contacts: a definition

**Figure 1:**
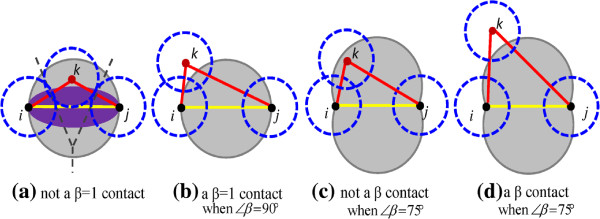
**Examples of*β* contacts and non-*β* contacts.** Three points, denoted by *i*, *j* and *k*, represent atoms. The dashed circles represent the van der Waals spheres in 2D space. The lines in yellow are of interest.

Given a quaternary structure of a protein complex *p*, a *β* contact between two atoms *i* and *j* in *p* requires that (i) the spatial distance between *i* and *j* is less than a threshold *T*_*d*_ plus the sum of their van der Waals radii defined by [30] (distance-cutoff contacts for short), (ii) *i* and *j* share a Voronoi facet in *p*’s Voronoi diagram, and (iii) the contact cannot break *p*’s *β*-skeleton. The *β*-skeleton [27] of a discrete set *p* is an undirected graph in computational geometry. In this graph, two points *i* and *j* have an edge if angle *ikj* is sharper than a threshold determined by *β*, ∀*k*∈*p*,*k*≠*i*,*j*. This angle threshold is denoted as *∠β*, which actually defines a forbidden region *fr* of the contact between *i* and *j*, e.g., the gray regions in Figure 1. When *β*=1, namely *∠β*=90°, *fr* is the sphere with the midpoint of *i* and *j* as the center and with *c*’s length as the diameter as shown in Figure 1(b). This sphere is similar to van der Waals radii of atoms. The forbidden region *fr* of a *β* contact usually does not cover any other atoms. Otherwise, if there is an atom *k* in *fr*, for example as shown in Figure 1(a) when *∠β*=90° or in Figure 1(c) when *∠β*=75°, the contact between *i* and *j* is not a *β* contact. A *β* contact suggests that its two atoms should have enough direct contact area to form an important interaction. The number of atomic *β* contacts in protein binding interfaces is only a small fraction number of distance-based contacts or less than half the number of contacts in the Voronoi diagrams when *T*_*d*_=3.3 as found by [28]. Interestingly, the use of *β* contacts can achieve better prediction performance for distinguishing false binding of crystal packing from homodimers.

#### A method to produce *β* contacts

A protein complex *p* can be represented as an atomic *β* contact graph *b*(*p*), if all of the heavy atoms are represented by nodes, and the *β* contacts are represented by edges. To produce *b*(*p*) for *p*, Qhull is first used to obtain the Delaunay triangulation [31] for all nodes. After that, the distance threshold *T*_*d*_ is used to remove those atomic contacts whose distances are too large. *T*_*d*_ is set as 3.3 Å (the diameter of a water molecule 2.8 Å plus 0.5 Å). This threshold is an insensitive factor to *β* contacts when it is large enough. Please refer to the Additional file 1 for an analysis of *β* contacts under several different *T*_*d*_s. Thirdly, each atomic contact is checked to guarantee that it satisfies the *β* skeleton requirements. To sharpen the difference of those mutations with higher *ΔΔG* and those with lower *ΔΔG*, the angle threshold *∠β* is set as 75° in this work, whose forbidden region *fr* is larger than that of *∠β*=90° as shown in Figure 1(b) and (c). That is, *∠β*=75° is a stricter condition than using *∠β*=90° to produce *β* contacts. The rationale to choose the stricter condition *∠β*=75° is illustrated in the following situation. Assume (i) A, B and C are the center points of three atoms with van der Waals radii 1.8 Å (for example, the radius of common Carbon atoms in protein structures), (ii) there is a covalent bond between A and B (AB for short) with spatial distance 1.5 Å, and a non-covalent bond between A and C (AC for short) and one between B and C (BC for short), and (iii) the van der Waals sphere of A and that of C are circumscribed each other with the spatial distance 3.6 Å, and the same for the van der Waals sphere of A and that of C. Then, the angle *∠ABC*=78°. A stricter threshold than 78° is 75°, which was chosen by this work.

### Our prediction methods

This section describes how to construct our *β*ACV_*ASA*_ classifier, including how to define *β*ACV and how to integrate *ASA* into *β*ACV.

#### *β*ACV: a vector representation for interfacial alanine mutations

We use an atomic contact vector (ACV) [32] of *β* contacts to represent an interfacial alanine mutation. We also use *β* contacts of the interfacial bound water molecules to update the vector, and use the atomic environment of the mutation to expand the basic vector.

##### Constructing a basic *β*ACV vector

To produce a basic *β*ACV for an alanine mutation *mutrala* of residue *r* in a protein complex *p*, we build the *β* contact graph *b*(*p*) for *p*. We then remove the coordinates of the mutated atoms in *r* to get a quaternary structure resulted from the mutation, denoted by *prmut*. Then, we produce another *β* contact graph *b*(*prmut*) for *prmut*.

By the mutation *mutrala*, some contacts in *b*(*p*) may disappear in *b*(*prmut*), namely those mutated contacts, while some new contacts can be formed in *b*(*prmut*), called new contacts. Both of those mutated and new contacts are represented in a *β*ACV vector. As the heavy atoms from the 20 standard residues are grouped into 8 atomic types (shown in Additional file 1: Table S4) by this work, our *β*ACV vector has 36 pairs of atomic types as elements. The value *v*(*Ti*,*Tj*) of each element in *β*ACV with atomic types *Ti* and *Tj* is calculated, using Equation 1.

(1) $$\begin{array}{ll} \;v(\text{Ti},\text{Tj})= & \underset{\left(x,y)\in M(\text{Ti},\text{Tj}\right)}{\sum }\frac{1}{d_{\left(x,y\right)}^{2}}\;-\underset{\left(x^{\prime },y^{\prime })\in N(\text{Ti},\text{Tj}\right)}{\sum }\frac{1}{d_{\left(x^{\prime },y^{\prime }\right)}^{2}}\; \end{array}$$

where *x* and *x*^′^ are of the atomic type *Ti*, *y* and *y*^′^ are of the atomic type *Tj*, (*x*,*y*) and (*x*^′^,*y*^′^) are two atomic pairs, *d*(∗,∗) is the spatial distance between a pair of atoms, and *M*(*Ti*,*Tj*)(or *N*(*Ti*,*Tj*)) is the set of all those mutated (or the set of all those new) contacts whose atomic types are *Ti* and *Tj*. Here term *d*^2^(∗,∗) is specially used to follow the same idea as Coulomb’s law which also uses the inverse of squared distance. Note that the other common contacts between *b*(*p*) and *b*(*prmut*) are not used in *β*ACV. Alanine mutations of Ala are assumed to have insignificant *ΔΔG* and alanine mutations of Gly are not considered.

It can be seen that a basic *β*ACV considers all mutated contacts and new contacts, including both acrossinterface contacts and those contacts from the same proteins or same biological units. However, atomic contacts between covalent-bond nearby atoms are not used in Equation 1. The covalent-bond nearby atoms of a given atom *i* are those atoms that have not more than three covalent-bond steps from *i*. For example, suppose *i*−*j*−*k*−*l*−*m*, where − indicates a covalent bond. From *i*, the covalent-bond step is 0 to *i*, is 1 to *j*, is 2 to *k*, is 3 to *l* and is 4 to *m*, respectively. Thus, *i*,*j*,*k* and *l* are covalent-bond nearby atoms of *i*, while *m* is not. In *β*ACV, the contacts between *i* and its covalent-bond nearby atoms are excluded from *M* or *N* in Equation 1. This is reasonable, because spatially close distances between *i* and its covalent-bond nearby atoms are more likely due to the rigidity of their covalent bonds.

##### Bound water molecules in protein interfaces

Protein folding and binding occur in a solvent environment *in vivo*. Water molecules are heavily involved in protein binding and sometimes they can form a compulsory part of the protein interfaces. In this work, a water molecule in PDB is considered as a part of a binding interface if (i) it has at least 3 potential hydrogen-bonds contacts, or (ii) it has 2 potential hydrogen-bond contacts and also has at least 2 other contacts with spatial distances less than 4 Å. A potential hydrogen-bond contact is required to have a spatial distance less than 3.2 Å between a hydrogen donor (such as a nitrogen atom) and a hydrogen acceptor (such as an oxygen atom). Water molecules under this requirement, named bound water molecules, are such closely involved in protein folding and binding that they can play an integral part. Bound water molecules are then grouped into the Oxygen atomic type with more than one hydrogen atom (shown in Additional file 1: Table S4) to update the values of the elements in the basic *β*ACV vector. We did not consider the contacts between any two water molecules.

##### The neighbourhood atoms of mutated residues

Information of neighbourhood atoms of *mutrala* is used to expand the basic *β*ACV vector. Assume that *S* is a set of atoms which have *β* contacts with the mutated atoms under *∠β*=90°. For each atom in *S* including the mutated atoms, its nearby atoms are added into *S*. Then, an atomic vector with the above 8 atomic types is also used to represent those atoms in *S* in the bound state. The value of its element *Tk* is calculated using *v*(*Tk*)^*b*^ in Equation 2. Similarly, *v*(*Tk*)^*u*^ in Equation 2 is used to calculate another atomic vector for representing the atoms in *S* in the unbound state.

(2) $$\;v{\left(\text{Tk}\right)}^{b}=\underset{j\in S,t_{j}=\text{Tk}}{\sum }E_{j}^{\text{loc}};\;\;\;v{\left(\text{Tk}\right)}^{u}=\underset{j\in S,t_{j}=\text{Tk}}{\sum }E_{j}^{\text{loc}}(u)$$

where *Ejloc* (or *Ejloc*(*u*)) is the relative local ASA of atom *j* in the bound (or unbound) state calculated via Equation 6 below. Water molecules were not considered here. Thus, each basic *β*ACV vector is now expanded by another 16 atomic types for representing surrounding information of mutated atoms. So, the expanded *β*ACV is a vector representation with 52 elements.

#### *β*ACV_*ASA*_: integrating the water exclusion hypothesis into *β*ACV

Solvent water is compulsory for protein binding, but water exclusion—small accessible surface area (ASA)—is a necessary condition for a residue to become binding hot spot [3,33,34]. Few highly exposed residues can make significant contribution to protein binding strength [34]. Thus, we integrate ASA information into each atomic pair of *β*ACV in Equation 1, and name the method *β*ACV_*ASA*_. We note that except Equation 1, the other definitions in *β*ACV_*ASA*_ are the same as those in *β*ACV.

Given a protein complex *p*, we take the following steps to integrate the water exclusion theory into Equation 1. The first step is to use NACCESS [30] to produce ASA for all of the atoms and residues in both bound and unbound states. For *p* in the bound state, we then define special ASA terms for an atom *i* using Equation 3, and for a residue *R*_*i*_ using Equation 4 and Equation 5.

(3) $$\begin{array}{llll} \;E_{i} & =\sqrt{\frac{{\text{ASA}}_{i}}{50}}\; & \;\;\;B_{i} & =\text{max}(0,1-E_{i})\; \end{array}$$

(4) $$\begin{array}{llll} \;E_{R_{i}}^{\text{bb}} & =\sqrt{\frac{{\text{ASA}}_{R_{i}}^{\text{bb}}}{\text{max}({\text{ASA}}_{R_{i}}^{\text{bb}})}}\; & \;\;\;B_{R_{i}}^{\text{bb}} & =\text{max}(0,1-E_{R_{i}}^{\text{bb}})\; \end{array}$$

(5) $$\begin{array}{llll} \;E_{R_{i}}^{\text{sc}} & =\sqrt{\frac{{\text{ASA}}_{R_{i}}^{\text{sc}}}{\text{max}({\text{ASA}}_{R_{i}}^{\text{sc}})}}\; & \;\;\;B_{R_{i}}^{\text{sc}} & =\text{max}(0,1-E_{R_{i}}^{\text{sc}})\; \end{array}$$

In Equation 3, *ASA*_*i*_ is accessible surface area of atom *i*, while *E*_*i*_ is its relative ASA, and *B*_*i*_ is its relative ASA burial compared to the maximum ASA, where number 50 is roughly half of NACCESS-calculated ASA of a single water molecule without any neighbor atoms (the ASA of a water molecule is 98.47=4×3.14×2.8^2^ Å^2^). In Equations 4 and 5, ${\text{ASA}}_{R_{i}}^{\text{bb}}$ and ${\text{ASA}}_{R_{i}}^{\text{sc}}$ are accessible surface area of backbone atoms (i.e., *bb*) and of side-chain atoms (i.e., *sc*) for a residue *R*_*i*_, while $E_{R_{i}}^{*}$ and $B_{R_{i}}^{*}$ are the relative ASA and the relative ASA burial of ∗∈{*bb*,*sc*}. $\text{max}({\text{ASA}}_{R_{i}}^{\text{bb}})$ and $\text{max}({\text{ASA}}_{R_{i}}^{\text{sc}})$ are the maximum ASA of backbone atoms and of side-chain atoms for the residue type of *R*_*i*_, which are calculated in a triplet of ALA- *R*_*i*_-ALA by NACCESS. These backbone atoms and side-chain atoms are defined in the same way as those in [30].

We compute the local ASA *Eiloc* and local ASA burial *Biloc* of an atom *i* via Equation 6.

(6) $$\begin{array}{lll} E_{i}^{\text{loc}} & =\left\{\begin{array}{c} E_{i}\times E_{R_{i}}^{\text{bb}}\;\text{if}i\text{is a backbone atom of}\;\;R_{i}\\ E_{i}\times E_{R_{i}}^{\text{sc}}\;\text{if}i\text{is a side-chain atom of}R_{i} \end{array}\right)\; & \;\\ B_{i}^{\text{loc}} & =\left\{\begin{array}{c} B_{i}\times B_{R_{i}}^{\text{bb}}\;\;\text{if}i\text{is a backbone atom of}\;\;R_{i}\\ B_{i}\times B_{R_{i}}^{\text{sc}}\;\text{if}i\text{is a side-chain atom of}\;\;R_{i} \end{array}\right)\; \end{array}$$

where the multiplication of relative ASA burial of both atom *i* and its residue is used to calculate local ASA burial *Biloc*. This is because relative ASA of both an atom and its residue are critical in describing the accessibility of an atom. For example, an atom may be buried with small ASA but its covalent-bond atoms might be exposed. When relative ASA of atoms or residues are used individually, the performance was worse (data not shown).

To integrate water exclusion theory into Equation 1, we determine the value *v*(*Ti*,*Tj*) of each element in *β*ACV_*ASA*_ through Equation 7 instead of Equation 1.

(7) $$\begin{array}{c} v(\text{Ti},\text{Tj})=\underset{\left(x,y)\in M(\text{Ti},\text{Tj}\right)}{\sum }\frac{B_{x}^{\text{loc}}\times B_{y}^{\text{loc}}}{d_{\left(x,y\right)}^{2}}\\ \;-\underset{\left(x^{\prime },y^{\prime })\in N(\text{Ti},\text{Tj}\right)}{\sum }\frac{B_{x^{\prime }}^{\text{loc}}\times B_{y^{\prime }}^{\text{loc}}}{d_{\left(x^{\prime },y^{\prime }\right)}^{2}} \end{array}$$

where *T*_∗_, *x*, *y*, *x*^′^, *y*^′^, *M* and *N* have the same meaning as those in Equation 1.

##### Comparison of *β* contacts with distance-cutoff contacts

To compare the performance of *β* contacts with distance-cutoff contacts for predicting *ΔΔG*, ACV_*ASA*_ based on distance-cutoff contacts is constructed in a similar way to constructing *β*ACV_*ASA*_. To further show the importance of *β* contacts in protein binding interfaces, non *β*ACV_*ASA*_ is also constructed for alanine mutations at the setting of *∠β*=90°. In non *β*ACV_*ASA*_, the values of its elements are the difference of the values of the 52 elements between ACV_*ASA*_ and *β*ACV_*ASA*_. To highlight the advantage of *β* contacts, ACV_*ASA*_ is also evaluated with different spatial distance thresholds (from 2.9 Å to 5 Å) for defining atomic contacts across interfaces and within binding sites.

Our *β*ACV_*ASA*_ classifier and its variants described above are summarized in Table 1.

**Table 1:** **Description of** ***β*** **ACV**_***ASA***_** and its variant methods**

**Methods**	**Description (of the representation for an**
	**alanine mutation)**
*β*ACV	An ACV of *β* contacts without ASA integration
*βACV*_*ASA*_	An ACV of *β* contacts with ASA integration
ACV_*ASA*_	An ACV of distance-cutoff contacts with ASA integration
non *βACV*_*ASA*_	The difference of *βACV*_*ASA*_ and ACV_*ASA*_

### Ridge regression: predict *ΔΔG* and binding hot spots

Ridge regression in Matlab is used here to learn a relation between atomic contact vectors and *ΔΔG*. By this regression, values in each column are normalized for the training dataset. Ridge regression minimizes average square error *SE* between the experimental ($\Delta \Delta G_{i}^{e}$) and the predicted $\Delta \Delta G_{i}^{p}$ in the training data with *N* mutations where $\text{SE}=\frac{\underset{i}{\sum }{\left(\Delta \Delta G_{i}^{e}-\Delta \Delta G_{i}^{p}\right)}^{2}}{N-1}$.

In our evaluation, leave-one-out cross-validation is used for all of the 396 mutations, and then the correlation coefficient *R* and average standard deviation $\delta =\sqrt{\text{SE}}$ are calculated. Under this evaluation framework, there is one outlier prediction by *βACV*_*ASA*_ and one outlier by *β*ACV for the whole dataset with 396 mutations. These outliers have less than -3 kcal/mol predicted *ΔΔG*, or more than 11 kcal/mol predicted *ΔΔG*, as shown in Additional file 1: Table S3. This may be due to limited alanine mutations of a high *ΔΔG* in the dataset.

In this work, predicted hot spot residues are those residue mutations with a predicted *ΔΔG*≥2 kcal/mol, same as the true hot spot definition.

### Hot spot prediction and evaluation measures

*β*ACV_*ASA*_ is also assessed by applying to the classification problem of binding hot spots. Classification performance is measured by *precision*(*p*.), *recall*(*r*.), *accuracy*(*acc*.) and *F*1 whose definitions are given in Equation 8.

(8) $$\begin{array}{c} \text{precision}(\mathrm{p.})=\frac{\text{TP}}{\text{TP}+\text{FP}}\\ \;\text{recall}(\mathrm{r.})=\frac{\text{TP}}{\text{TP}+\text{FN}}\\ \;\text{accuracy}(\mathrm{acc.})=\frac{\text{TP}+\text{TN}}{\text{TP}+\text{TN}+\text{FP}+\text{FN}}\\ \;F1=\frac{2\times \text{precision}\times \text{recall}}{\text{precision}+\text{recall}} \end{array}$$

where binding hot spots are considered as the true cases, while non-hot spots as the false cases; TP, FP, TN and FN are true positives, false positives, true negatives and false negatives, respectively. Hence, *precision* is the number of correct hot spot predictions divided by the number of positive predictions, *recall* is the fraction of correct hot spot predictions over all hot spots, while *accuracy* is the number of correctly predicted hot spots and non-hot spots divided by the number of all mutations. These measures are also used in [14,20,35] with the same definitions.

## Results and discussion

### *β* contacts are better than distance-cutoff contacts for predicting *ΔΔG*

**Figure 2:**
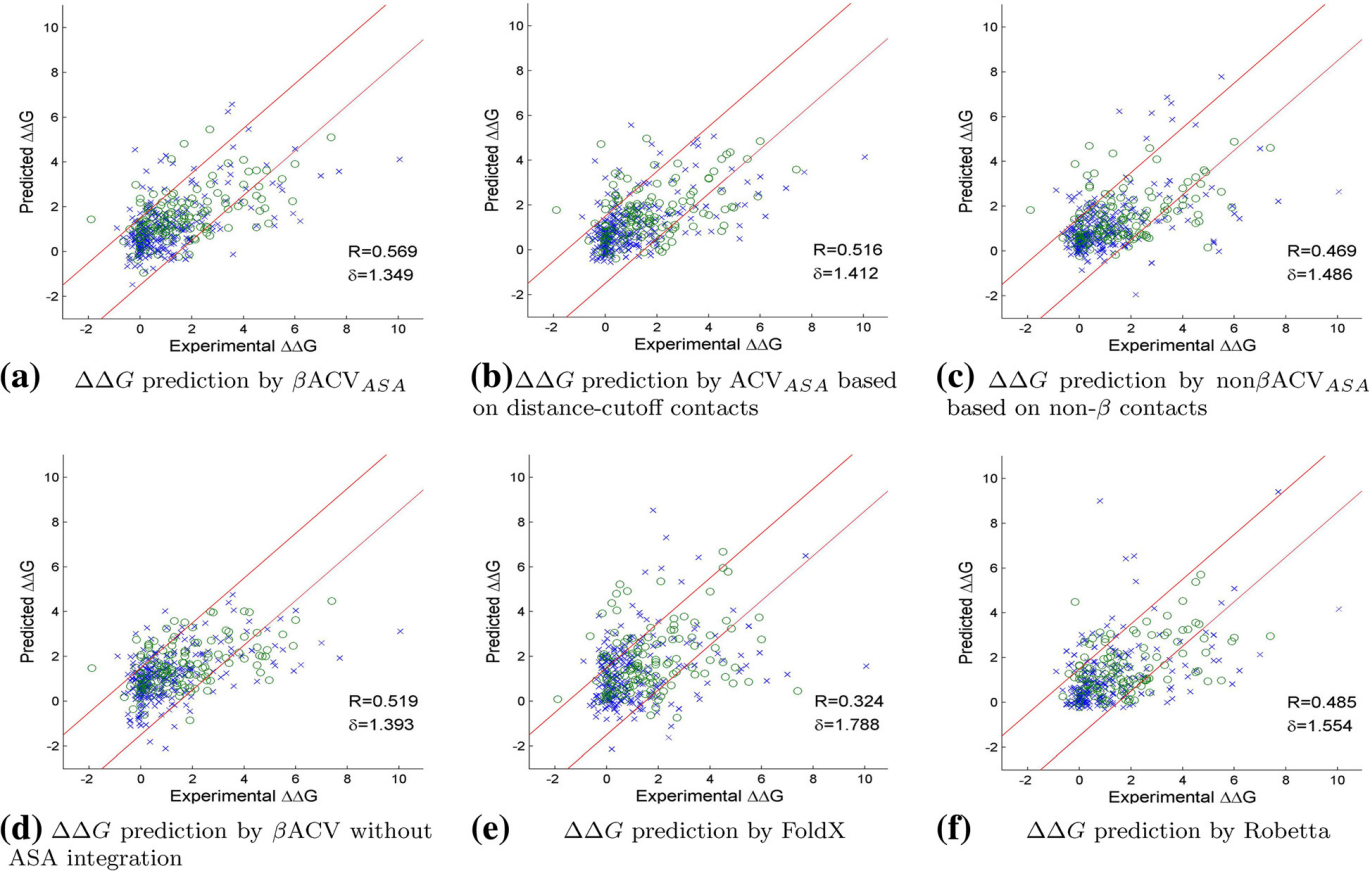
***ΔΔG* predicted by different methods.** In **(a)-(d)**, ‘o’ represents non-polar residues, while ‘x’ represents polar residues. *R* is the Pearson correlation coefficient and *δ* is the average standard deviation. *R* is specially calculated for **(a)** and **(d)** based on the data set after removing the one outlier prediction. *R* is slightly changed to 0.543 or 0.515 respectively when the outlier is not removed. The two diagonal red lines represent *y*=*x*±1.5.

Our *βACV*_*ASA*_ classifier is compared with ACV_*ASA*_ and with non *β*ACV_*ASA*_ to show the importance of *β* contacts in the prediction of *ΔΔG* under alanine mutations. The prediction results are presented in Figure 2(a), (b) and (c).

It can be seen from Figure 2(a) and (b) that *β*ACV_*ASA*_ has a better *ΔΔG* prediction performance according to both correlation coefficient *R* and average standard deviation *δ*. The number of *β* contacts used by *β*ACV_*ASA*_ is only a small fraction of the number of distance-cutoff contacts used by ACV_*ASA*_. For example, there are 54,286 distance-cutoff contacts across binding interfaces for the 22 protein complexes, but there are only 9,830 *β* contacts across the binding interfaces (*∠β*=90°), and 4,096 *β* contacts under the setting of *∠β*=75° which is actually used by *β*ACV_*ASA*_. So, *β*ACV_*ASA*_ uses only 7.55% number of distance-cutoff atomic contacts but it achieves a better prediction performance.

The comparison between *β*ACV_*ASA*_ and non *β*ACV_*ASA*_ (Figure 2(a) and (c)) further suggests the importance of *β* contacts in *ΔΔG* prediction. In Figure 2(c), non *β*ACV_*ASA*_ has much lower *R* (0.469) and a higher *δ* (1.486) than *β*ACV_*ASA*_, but non *β*ACV_*ASA*_ uses all non- *β* contacts of *∠β*=90°, that is, 81.8% number of distance-cutoff atomic contacts.

A lot of alanine mutations are not binding hot spot residues, having a small *ΔΔG*, i.e., <2 kcal/mol. These mutations heavily affect the calculation of *R* and *δ*. On the other hand, the prediction of residue mutations with a high *ΔΔG* is more important. Thus, the classification performance for these binding hot spots is also assessed. The results are shown in Table 2. It is noted that F1 is not the objective function to be optimized in the regression process.

**Table 2:** Prediction performances by different methods for the same set of binding hot spots

**Methods**	**Precision**	**Recall**	**F1**	**Accuracy**
*β*ACV_*ASA*_	0.615	0.593	0.604	0.830
ACV_*ASA*_	0.526	0.477	0.500	0.793
non *βACV*_*ASA*_	0.513	0.454	0.482	0.788
*β*ACV	0.564	0.616	0.589	0.813
FoldX	0.400	0.488	0.440	0.730
Robetta	0.526	0.465	0.494	0.793
HotPOINT	0.439	0.547	0.487	0.750
KFC2a	0.443	0.767	0.562	0.740
KFC2b	0.521	0.570	0.544	0.793

In Table 2 among *β*ACV_*ASA*_, ACV_*ASA*_ and non *β*ACV_*ASA*_, *β*ACV_*ASA*_ has the highest precision, recall, F1 and accuracy, while non *β*ACV_*ASA*_ has the lowest. For example, *β*ACV_*ASA*_’s F1 is 0.604, 0.122 higher than non *β*ACV_*ASA*_’s F1. However, ACV_*ASA*_ and non *β*ACV_*ASA*_ show quite similar performances. The reason would be that non- *β* contacts (used by non *β*ACV_*ASA*_) are often dominant in distance-cutoff contacts (used by ACV_*ASA*_).

The performances of ACV_*ASA*_ with different spatial distance thresholds are shown in Table 3. We can see that the performance has a growing tendency when the threshold increases. Nevertheless, the best performance of ACV_*ASA*_ (*F*1 = 0.5 in Table 2) is much lower than that of *β*ACV_*ASA*_ (*F*1 = 0.604). Of special interest, when the spatial distance threshold is set at 3.6 Å, ACV_*ASA*_ has a number of distance-cutoff contacts nearly the same as the number of our used *β* contacts. In this special case of almost the same number of contacts used, ACV_*ASA*_ has much worse performance than *β*ACV_*ASA*_, and only about half of the distance-based contacts are *β* contacts across the 22 protein-protein binding interfaces. These results affirm that *β* contacts are advantageous over distance-based contacts for predicting binding hot spot residues.

**Table 3:** **Prediction performance and the numbers of used contacts by** ***β*** **ACV**_***ASA***_** and ACV**_***ASA***_

**Methods**	**Distance**^**1**^	**#contacts**^**2**^	**Precision**	**Recall**	**F1**	**Accuracy**
***β*** **ACV**_***ASA***_		**2,881**	**0.615**	**0.593**	**0.604**	**0.830**
ACV_*ASA*_	2.9	347	0.486	0.419	0.450	0.778
	3.0	513	0.465	0.382	0.420	0.770
	3.1	715	0.394	0.302	0.342	0.747
	3.2	966	0.487	0.442	0.463	0.778
	3.3	1,293	0.450	0.419	0.434	0.763
	3.42	1,884	0.438	0.372	0.403	0.760
	3.5	2,394	0.494	0.442	0.466	0.780
	3.55	2,789	0.443	0.407	0.424	0.760
	3.6	3,123	0.437	0.360	0.395	0.760
	4	7,542	0.463	0.430	0.446	0.768
	4.5	15,389	0.482	0.465	0.473	0.775
	5	26,752	0.488	0.465	0.476	0.778

^1^: The spatial distance threshold of two atoms.

^2^: The number of atomic contacts involving in the 396 mutations, including mutated contacts and new contacts.

### Water exclusion is a necessary condition of hot spot binding

Literature work has reported that water exclusion is a necessary condition for an interfacial residue to become a hot spot residue [3,33]. To confirm the importance of water exclusion in the prediction of *ΔΔG*, the performance by *β*ACV when ASA is not integrated is assessed. This prediction performance is shown in Figure 2(d) and Table 2. Comparing Figure 2(d) with Figure 2(a), *β*ACV_*ASA*_ has a better regression performance than *β*ACV indeed. It also has a better F1 performance as seen in Table 2. These results confirm that water exclusion plays an important role in hot spot prediction, and it should be a necessary condition for an interfacial residue to become a binding hot spot residue in protein-protein complexes.

### Our method *β*ACV_*ASA*_ is superior to several widely-used methods

Our *β*ACV_*ASA*_ classifier is also assessed against the state-of-the-art methods FoldX [9,10], Robetta [11], HotPOINT [20] and KFC [36]. The prediction performances of these previous methods were obtained through their web servers (Robetta, HotPOINT and KFC) or the standalone executable program (FoldX with default settings).

#### Comparison results

Figures 2(e) and 2(f) show that the prediction performance of FoldX and Robetta are much worse than our *β*ACV_*ASA*_. These two methods have a *R* of 0.324 or 0.485, much smaller than *β*ACV_*ASA*_’s 0.569; their *δ* is 1.788 or 1.554, much larger than *β*ACV_*ASA*_’s 1.349. Table 2 also shows their classification performance on the 396 mutations: FoldX’s F1 is 0.44, while Robetta’s F1 is 0.494, both worse than *β*ACV_*ASA*_’s 0.604. From Table 2, our method also has better classification performance than HotPOINT and KFC. Other performance comparison results are provided in the Additional file 1 when tested on BID (including protein-peptide interfacial residues) or under the leave-one-complex-out cross-validation framework.

#### An example of hot spot predictions

We use 3HFM as a case study to illustrate the difference of the binding hot spot prediction results by *β*ACV_*ASA*_, FoldX and Robetta. The 3HFM complex is an antibody-antigen binding between HyHEL-10 and hen egg white lysozyme. According to ASEdb, a total of 25 alanine mutations were experimented, 11 of which have *ΔΔG* more than 2 kcal/mol.

**Figure 3:**
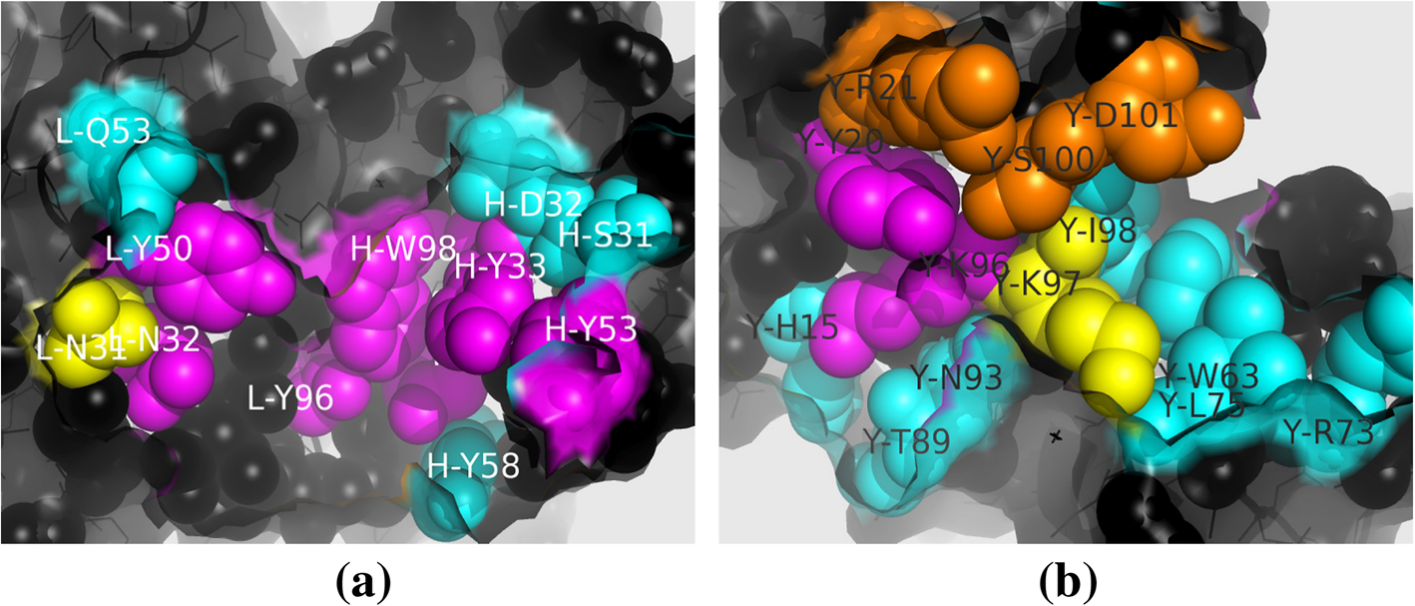
**Prediction results by*β*ACV**_***ASA***_** for the residues in the interface between Chain Y and Chain HL(together) in 3HFM.** In **(a)** and **(b)**, the true predicted hot spot residues are in magenta, the false predicted non-hot spot residues are in yellow, the false predicted hot spot residues are in orange, while the true predicted non-hot spots are in cyan; X-YZZ stands for residue Y in position ZZ of Chain X.

Our *β*ACV_*ASA*_ correctly identified 9 binding hot spot residues with a recall of 0.818, but made 3 false positive predictions with a precision of 0.75 (Figure 3). This gives an F1 of 0.783. However, FoldX made only one hot spot prediction which is correct with a recall of 0.091, and Robetta has a recall of 0.455 (5 out of 11) and a precision of 0.833 (5 out of 6), namely an F1 of 0.588. Both of these methods have a lower prediction performance than our *β*ACV_*ASA*_. What is more important is that the four positive predictions correctly made only by our *β*ACV_*ASA*_, not by FoldX or Robetta, have a high *ΔΔG*, such as Trp in position 98 of Chain H (*ΔΔG*=5.5 kcal/mol) and Tyr in position 50 of Chain L (*ΔΔG*=4.6 kcal/mol). Please refer to Additional file 1: Table S3 for detail.

## Conclusion

A new classifier *β*ACV_*ASA*_ has been proposed to predict *ΔΔG* and binding hot spot residues. The novel idea of this classifier is to integrate the water exclusion theory into *β* contacts. Tested on a data set of 396 alanine mutations, *β*ACV_*ASA*_ has been found to outperform many other methods. This confirms that *β* contacts are truly better than traditional distance-cutoff contacts to capture the energetic characteristics of protein folding and binding. This also confirms that water exclusion is a necessary condition for a residue to become a binding hot spot residue.

## Availability of supporting data

All the supporting data are included as additional files.

## Competing interests

The authors declare that they have no competing interests.

## Authors’ contributions

QL designed the methods and performed the experiments. JL and SH supervised the study. JL, LW and CK participated in the analysis. QL drafted the manuscript. QL, SH, LW, CK and JL read and revised the manuscript. All authors approved the final version.

## Supplementary Material

Additional file 1 **This additional file covers an analysis on** ***β* contacts of different** ***T***_***d***_**s, more evaluation results and related discussions (including the statistical significance of the difference among Figure**2**(a) to**2**(d), the dataset and evaluation on BID, the evaluation under leave-one-complex-out cross-validation, and a discussion on using the 396 mutations), the groups of atomic types, and the detail of our dataset.** Click here for file
