# Supplementary material for: Integrating water exclusion theory into βcontacts to predict binding free energy changes and binding hot spots
Source: BMC Bioinformatics. 2014 Feb 26;15:57. doi: 10.1186/1471-2105-15-57 (PMC3941611; doi:10.1186/1471-2105-15-57)
Supplement: Additional file 1 — This additional file covers an analysis on β contacts of different T d s, more evaluation results and related discussions (including the statistical significance of the difference among Figure2(a) to2(d), the dataset and evaluation on BID, the evaluation under leave-one-complex-out cross-validation, and a discussion on using the 396 mutations), the groups of atomic types, and the detail of our dataset. [file 1471-2105-15-57-S1.pdf]

Prediction of binding free energy and binding hot spots by integrating water exclusion into β atomic contacts — supplementary

Qian Liu [1,2], Steven C.H. Hoi [2], Chee Keong Kwoh[2], Limsoon Wong[3] and Jinyan Li[*,1]

[1]Advanced Analytics Institute and Center for Health Technologies, Faculty of Engineering and IT, University of Technology Sydney, Australia
[2]School of Computer Engineering, Nanyang Technological University, Singapore 639798
[3]School of Computing, National University of Singapore, Singapore 117417

Email: Qian Liu - qian.liu@uts.edu.au; Steven C.H. Hoi - chhoi@ntu.edu.sg; Chee Keong Kwoh - asckkwoh@ntu.edu.sg; Limsoon Wong - dcswls@nus.edu.sg; Jinyan Li - jinyan.li@uts.edu.au;

*Corresponding author

1 An Analysis of β Contacts Under Different T_ds

T_d has no significant impact on β contacts if large enough. For example, under $\angle\beta = 75^\circ$ and in the 396 mutation residues (including backbone atoms), there are 32 involving β contacts whose distances are between 2.8 Å and 3.3 Å, 118 β contacts with distances between 1.9 Å and 2.8 Å, and 5760 β contacts whose distance less than 1.9 Å. Here, 1.9 Å is the radius of a water molecule plus 0.5 Å. The latter contacts with small distance predominate in β contacts.

2 Discussion of the evaluation

2.1 Statistical Significance of Difference Among Figure 2(a)-2(c)

Paired t-test in MATLAB is used to test the difference among Figure 2(a)-2(d) for the predictions of true hot spots. This test shows that the p-value of the difference of these predictions between Figure 2(a) and 2(b), between Figure 2(a) and 2(c), between Figure 2(a) and 2(d), and between Figure 2(b) and 2(c) are 0.016, 0.108, 0.010 and 0.798, respectively. Figure 2(a) of βACV$_{ASA}$ predictions is more significantly different from the other figures, while Figure 2(b) of ACV$_{ASA}$ predictions and 2(c) of nonβACV$_{ASA}$ predictions have insignificant difference.

2.2 Evaluation under leave-one-complex-out cross-validation

The performance of βACV$_{ASA}$ and ACV$_{ASA}$ are also evaluated under the leave-one-complex-out cross-validation where those mutations in each of 22 protein-protein complexes are used for testing and other mutations for training. Under this evaluation strategy, βACV$_{ASA}$ has a F1 value 0.588 with a precision 0.595 and a recall 0.581, and ACV$_{ASA}$ has a F1 value 0.454 with a precision 0.481 and a recall 0.430. This performance slightly decreases compared with the leave-one-out cross-validation. But the small performance decrease is insignificant, and all the conclusions above are still true using the performance of leave-one-complex-out cross-validation: βACV$_{ASA}$ is still superior to other methods to predict $\Delta\Delta G$. Meanwhile, the small performance decrease is reasonable because the number of mutations are not big and leave-one-complex-out cross-validation has less mutations in training process.

2.3 Discussion of using the 396 mutations

The benchmark dataset used by this work consists of 396 residues. However, under the definition of binding interfaces by FoldX, only 378 of them are interfacial residues. Therefore, FoldX could make only 378 predictions for alanine mutations. Similar situation happened to Robetta which made only 338 predictions for the same dataset of 396 residues (details shown in Supplementary Table 3). In fact, some of those residues not detected by FoldX or Robetta are actually in the rim of binding interfaces (rim mutations for short), and the other have close contacts with their partner proteins, such as the two mutations of Pro in position 306 of Chain B in 1A22 and Glu in position 80 of Chain M in 1DX5. Although these two mutation residues are not defined to be in binding interfaces by FoldX and/or Robetta, their smallest spatial distance are less than 6Å to the atoms in their partner proteins. These two mutations can also affect protein binding significantly: the alanine mutation of Pro has $\Delta\Delta G$ 3.31 kcal/mol, and the alanine mutation of Glu has $\Delta\Delta G$ 3.4 kcal/mol.

If these two residue mutations were considered to have a small predicted $\Delta\Delta G$, Robetta would have a lower regression performance. Nevertheless, only the detected mutations by FoldX and Robetta are used in their regression performance assessment. In the classification performance comparison, all 396 mutations are used, and all non-predicted mutations by every classifier are considered to have a small predicted $\Delta\Delta G$. This is fair to all of the classifiers.

3 Dataset from BID and the evaluation

The mutations from BID [1] are also used for independent data testing. Those mutations have no explicit $\Delta\Delta G$ but with the class labels 'Strong', 'Intermediate', 'Weak','Insignificant' and so on. Often, the mutations with 'Strong' are considered as binding hot spots. In BID, we found 22 complexes. However, many of them are protein binding to a short sequence of peptides, such as with less than 20 residues in Chain E in 1CDL, B in 1DDM, X in 1dVA, C and D in 1EBP and D in 1JPP, and with less than 40 residues in A in 1DX5, and P in 1K4U. Protein-peptide binding complexes are not considered in this work, because they have different physicochemical properties. Meanwhile, some other complexes have very small interfaces (1IHB and 1KTZ) or have mutations in linear peptide structures (1G3I and 1UB4). They are also not considered because small interfaces have great effect on interfacial residues' ASA. 1GL4 is also not used due to the big difference of the definition of binding hot spots between BID data and the work in [2]. Finally, only protein-protein complexes are used for testing, including 1ES7, 1FAK, 1FE8, 1FOE, 1JAT, 1MQ8, 1NFI, 1NUN and 2HHB. This dataset only has 37 mutations with 7 binding hot spots, called BID-propro.

Finally, the prediction results on BID are shown in Table 1. It can be seen from Table 1 that on the small BID dataset BID-propro, βACV_{ASA} has F1 value 0.571, higher than FoldX's F1 0.480 and Robetta's F1 0.556. βACV_{ASA} achieves better performance than the existing methods again, although the number of the binding hot spots is too small to have a significant evaluation. Table 1 also shows the prediction results on both protein-protein complexes and protein-peptide complexes in BID with two different binding hot spot definitions. If only the mutations with the label 'Strong' are considered as binding hot spots, FoldX has highest F1 value, while Robetta and βACV_{ASA} have higher F1 values if the mutations with the label 'Strong' or 'Intermediate' are considered as binding hot spots. According the difference between the performances on BID-propro and BID, it seems that βACV_{ASA} is better to be used to predict binding hot spots for protein-protein complexes than for protein-peptide complexes. This is partially due to that ASA used in βACV_{ASA} affects the prediction performance, but ASA of interfacial residues in protein-protein complexes is different from that from protein-peptide complexes (interfacial residues of protein-peptide complexes might be more exposed than interfacial residues in protein-protein complexes). The reason is supported by the improved performance of βACV on BID where ASA is not used.

Table 1: **Predictions performance by different methods for protein complexes in BID.**

Data set	True hot spot labels	F1			
		FoldX	Robetta	βACV$_{ASA}$	βACV
BID-propro	Strong	0.480	0.556	0.571	0.533
BID	Strong	0.330	0.288	0.294	0.318
BID	Strong, Intermediate	0.562	0.627	0.626	0.641

Table 2: The used dataset.

PDB ID	Biological Unit 1			Biological Unit 2		
	Chain Name(s)	mutations	≥ 2 kcal/mol	Chain Name(s)	mutations	≥ 2 kcal/mol
1A22[1]	growth hormone			growth hormone receptor		
	A	30	3	B	32	5
1A4Y[4]	ribonuclease inhibitor			angiogenin		
	A	13	2	B	11	1
1AHW[5]	immunoglobulin fab 5g9			tissue factor		
	AB	0	0	C	8	1
1BRS	barnase			barstar		
	A	7	6	D	5	3
1BXI	colicin e9 immunity			colicin e9		
	A	17	7	B	0	0
1CBW[6]	bovine chymotrypsin			BPTI		
	GH	0	0	I	8	0
1DAN[5]	blood coagulation factor VIIA			soluble tissue factor		
	HL	25	0	TU	35	2
1DFJ[4]	ribonuclease			ribonuclease inhibitor		
	E	0	0	I	13	4
1DVF[3]	FV D1.3			FV E5.2		
	AB	16	6	CD	8	3
1DX5	thrombomodulin			thrombin		
	I	0	0	M	16	5
1F47	cell division protein ftsz			cell division protein zipa		
	A	9	3	B	0	0
1FC2	protein A			immunoglobulin fc		
	C	3	1	D	0	0
1FCC	IGG1 MO61 FC			streptococcal protein G		
	A	0	0	C	8	4
1GC1	CD4			envelope protein GP120		
	C	17	0	G	0	0
1JCK	T cell antigen receptor			staphylococcal enterotoxin C3		
	A	0	0	B	9	4
1JRH	antibody A6			interferon-gamma receptor alpha chain		
	HL	17	3	I	12	5
1JTG	beta-lactamase TEM			beta-lactamase inhibitory protein		
	A	6	0	B	4	2
1NMB	Fab NC10			N9 neuraminidase		
	HL	1	0	N	0	0

Continued on next page

Table 2 – continued from previous page

PDB ID	Biological Unit 1			Biological Unit 2		
	Chain Name(s)	mutations	≥ 2 kcal/mol	Chain Name(s)	mutations	≥ 2 kcal/mol
1VFB[2,3]	IGG1-KAPPA D1.3 FV			hen egg white lysozyme		
	AB	14	2	C	12	1
2PTC[6]	beta-trypsin			trypsin inhibitor		
	E	0	0	I	2	2
3HFM[2]	Hyhel-10 IGG1			hen egg white lysozyme		
	HL	12	8	Y	13	3
3HHR[1]	human growth hormone			human growth hormone receptor		
	A	10	0	BC	3	0

[1]: These two complexes have identical protein sequences, but they have different alanine mutations. Please refer to Supplementary Table 2 for detail.

[2]: Chain C in 1VFB and Chain Y in 3HFM are identical, but they have different binding sites in 1VFB and in 3HFM.

[3]: Chain A and B in 1VFB and in 1DVF are identical, but their partner proteins Chain C in 1VFB and Chain C and D in 1DVF are dissimilar, indicating that 1VFB and 1DVF possess different interfaces in 3D structures. $\Delta\Delta G$ of alanine mutations for the same specific residues in Chain A and B are almost different. In detail, $\Delta\Delta G$ for the same mutations in Chain A are: His^{130}:0.8(1.7), Tyr^{32}:1.3(2), Tyr^{49}:0.8(1.7), Tyr^{50}:0.4(0.7), Trp^{92}:1.71(0.3) and Ser^{93}:0.11(1.2); $\Delta\Delta G$ for the same mutations in Chain B are: Tyr^{32}:0.5(1.8), Trp^{52}:1.23(4.2), Asp^{54}:1.95(4.3), Asp^{58}:-0.2(1.6), Arg^{99}:0.47(1.9), Asp^{100}:3.1(2.8) and Tyr^{101}:4(4); the numbers after ':' are $\Delta\Delta G$ in 1VFB, and the numbers in brackets are $\Delta\Delta G$ in 1DVF.

[4]: Chain B in 1A4Y has dissimilar sequence to Chain E in 1DFJ, and Chain A in 1A4Y is similar to Chain I in 1DFJ. The latter two similar sequences have several same mutations but with different $\Delta\Delta G$ as follows: Trp^{261}:0.1(^{257}:1.3), Trp^{263}:1.2(^{259}:2.2), Ser^{289}:0(^{285}:0.8)), Trp^{318}:1.5(^{314}:1), Lys^{320}:-0.3(^{316}:1.3), Glu^{401}:0.9(^{397}:1.3)), Tyr^{434}:3.3(^{430}:5.9)), Asp^{435}:3.5 (^{431}:3.6)), Tyr^{437}:0.8(^{433}:2.6)) and Ile^{459}:0.7(^{455}:0.3)). The numbers after ':' are $\Delta\Delta G$ in 1A4Y and those in brackets are $\Delta\Delta G$ in 1DFJ.

[5]: Chain T and U in 1DAN constitute Chain C in 1AHW, but Chain A and B in 1AHW have different sequences from Chain H and L in 1DAN. Chain C in 1AHW (Chain T and U in 1DAN) also has different binding sites in these two complexes.

[6]: Chain I in 2PTC and in 1CBW are identical, but Chain E in 2PTC has 42% sequence identity to Chain G in 1CBW and 44% to Chain H in 1CBW. Further, Chain I in 2PTC only has two mutations: Lys^{15} and Ile^{18} which has $\Delta\Delta G$ 10 kcal/mol and 4.97 kcal/mol. In contrast, their mutations have much smaller $\Delta\Delta G$ in 1CBW, i.e., 1.99 kcal/mol and 1.4 kcal/mol.

Table 3: Alanine mutations in the used dataset. 'Inter' denotes the interaction of two biological units split by '-', and 'C' indicates protein chain names. ^{a} and ^{sc} represent the ASA change of all atoms from a residue or of side-chain atoms, while ^{ad} and ^{m} denote the surface distance d of those contacts of all atoms from a residue or of the mutated atoms; d is the Euclidian distance of two atoms minus the sum of their van der Waals radii as defined in [3]; $\underline{\vee}$ or $\overline{\wedge}$ indicates an outlier prediction with predicted $\Delta\Delta G$ less than -3 kcal/mol or more than 11 kcal/mol.

PDB	Inter	C	Pos	AA	$\Delta\Delta G_{exp}$	ASA^{a}	ASA^{sc}	d^{ad}	d^{m}	FoldX	Robetta	βACV$_{ASA}$	βACV$^{2}_{ASA}$
1A22	A-B	A	18	HIS	-0.5	71.67	71.66	-0.47	-0.47	-0.220	**2.290**	0.402	-0.420
	A-B	A	21	HIS	0.2	31.11	31.11	-0.15	-0.15	0.190	0.830	-0.284	-0.756
	A-B	A	22	GLN	-0.2	16.35	15.83	0.13	0.40	-0.060	0.050	0.372	0.410
	A-B	A	25	PHE	-0.4	47.89	47.89	0.12	0.12	0.920	1.290	1.005	-0.861
	A-B	A	26	ASP	-0.2	0.00	0.00	2.93	2.93	-0.040	-	0.379	0.610
	A-B	A	42	TYR	0.2	78.51	78.51	-0.05	-0.05	0.940	**2.020**	1.016	1.297
	A-B	A	45	LEU	1.2	68.67	52.10	-0.40	0.04	1.430	1.150	1.762	1.860
	A-B	A	46	GLN	0.1	76.77	71.59	-0.30	-0.08	-0.220	1.040	0.608	0.649
	A-B	A	48	PRO	0.4	57.51	57.50	-0.04	-0.04	1.090	-	0.485	1.433
	A-B	A	51	SER	0.3	10.78	8.02	1.24	1.34	-0.360	-0.020	1.081	0.436
	A-B	A	56	GLU	0.4	20.98	20.98	-0.17	-0.17	1.120	0.970	**2.796**	**3.076**
	A-B	A	61	PRO	1.2	6.41	0.00	0.51	2.15	0.290	-	0.409	0.686
	A-B	A	62	SER	0.1	74.12	42.31	-0.34	1.02	0.190	-0.170	1.004	1.052
	A-B	A	63	ASN	0.3	41.34	38.72	0.15	0.95	0.240	0.390	1.135	1.782
	A-B	A	64	ARG	1.6	122.01	115.92	-0.15	-0.15	1.280	**2.000**	1.181	1.631
	A-B	A	65	GLU	-0.5	23.68	23.68	1.01	1.01	0.010	-0.110	-0.657	-0.582
	A-B	A	66	GLU	0.43	0.00	0.00	2.76	2.76	0.890	-	0.851	0.991
	A-B	A	68	GLN	0.6	37.83	37.83	-0.07	-0.07	1.620	1.810	1.684	1.403
	A-B	A	164	TYR	0.3	22.41	22.41	1.14	1.14	1.780	-	**2.022**	-0.745
	A-B	A	167	ARG	0.3	34.74	34.74	-0.01	-0.01	1.190	0.420	1.724	1.331
	A-B	A	168	LYS	-0.2	50.24	49.83	0.10	0.10	**2.090**	**2.120**	**4.543**	**4.814**
	A-B	A	171	ASP	0.8	58.04	57.55	0.14	0.14	1.490	**9.000**	**4.044**	**3.666**
	A-B	A	172	LYS	**2**	30.23	30.23	0.21	0.21	**2.540**	0.740	1.227	1.490
	A-B	A	174	GLU	-0.9	11.59	11.59	0.60	0.60	1.790	1.380	1.057	0.362
	A-B	A	175	THR	**2**	48.25	48.11	-0.40	-0.40	**3.570**	**2.310**	**3.198**	**2.812**
	A-B	A	176	PHE	1.9	6.85	6.85	0.52	0.52	1.860	-	**2.174**	**3.074**

Continued on next page

Table 3 – continued from previous page

PDB	Inter	C	Pos	AA	$\Delta\Delta G_{exp}$	ASA^a	ASA^{sc}	d^{ad}	d^m	FoldX	Robetta	βACV$_{ASA}$	βACV$^2_{ASA}$
	A-B	A	178	ARG	**2.4**	61.29	61.29	-0.07	-0.07	1.900	0.380	1.307	0.957
	A-B	A	179	ILE	0.8	22.30	22.14	0.21	0.21	**2.800**	1.000	1.557	0.589
	A-B	A	182	CYS	1.01	32.49	32.27	-0.19	-0.19	1.490	-0.070	1.267	0.779
	A-B	A	183	ARG	0.5	2.50	2.50	2.32	2.32	1.960	-	-0.756	-0.521
	A-B	B	243	ARG	**2.12**	30.86	30.48	-0.40	-0.40	**5.930**	**6.540**	**2.179**	**2.179**
	A-B	B	244	GLU	1.69	32.18	32.18	0.01	0.01	**2.050**	0.490	0.941	0.736
	A-B	B	270	ARG	0.69	0.40	0.40	2.31	2.31	**2.120**	-	0.099	0.043
	A-B	B	271	ARG	0.54	75.59	75.59	-0.31	-0.31	**2.290**	1.310	$\veebar$	$\veebar$
	A-B	B	274	GLN	0	30.70	14.25	0.56	1.68	**2.100**	0.030	0.143	0.200
	A-B	B	275	GLU	-0.1	21.98	21.98	0.11	0.11	1.990	0.090	-0.242	0.074
	A-B	B	276	TRP	0.51	56.88	56.87	-0.40	-0.40	**5.220**	**2.860**	**2.572**	$\veebar$
	A-B	B	280	TRP	-0.02	3.85	3.85	1.88	1.88	**2.000**	-	-0.015	0.015
	A-B	B	298	SER	-0.05	22.82	12.65	1.01	2.55	**2.000**	-	0.298	0.353
	A-B	B	301	THR	1.76	12.52	0.00	0.66	2.75	-	-	0.031	-1.290
	A-B	B	302	SER	-0.2	24.90	24.90	0.23	0.95	1.960	-0.110	0.944	1.107
	A-B	B	303	ILE	1.61	18.56	7.78	-0.34	0.36	**2.860**	0.310	**2.207**	$\veebar$
	A-B	B	304	TRP	**4.5**	154.87	135.22	0.10	0.15	**5.960**	**5.380**	**3.626**	$\veebar$
	A-B	B	305	ILE	1.94	13.13	13.13	0.50	0.50	**2.560**	0.130	1.020	-1.275
	A-B	B	306	PRO	**3.31**	36.26	36.26	0.04	0.49	**2.710**	-	**2.263**	**3.022**
	A-B	B	320	GLU	-0.19	39.73	31.76	-0.00	-0.00	1.910	0.670	0.726	0.691
	A-B	B	321	LYS	0.08	16.18	16.18	0.45	0.79	**2.440**	-	1.265	1.408
	A-B	B	324	SER	0.28	11.38	11.38	1.36	1.67	**2.370**	-0.060	0.485	1.073
	A-B	B	326	ASP	0.99	14.49	14.49	0.61	1.41	**2.050**	-0.310	1.446	1.220
	A-B	B	327	GLU	0.97	63.40	63.40	-0.01	-0.01	1.860	1.110	1.719	0.430
	A-B	B	364	ASP	1.49	19.02	19.02	-0.15	-0.15	**5.770**	1.370	1.598	0.923
	A-B	B	365	ILE	**2.13**	29.47	17.32	0.44	1.30	**2.750**	0.090	0.537	0.716
	A-B	B	366	GLN	0.02	35.46	17.83	0.50	2.12	**2.800**	0.680	0.333	0.246
	A-B	B	367	LYS	-0.02	66.29	35.17	-0.11	-0.11	1.780	0.220	0.462	0.732
	A-B	B	369	TRP	**4.5**	92.93	91.27	-0.05	-0.05	**6.670**	**3.560**	**2.497**	**4.597**
	A-B	B	371	VAL	-0.64	36.84	36.84	0.17	0.17	**2.910**	0.260	0.459	-0.028
	A-B	B	394	THR	0.2	0.00	0.00	3.23	3.23	-	-	-0.875	-0.840
	A-B	B	395	THR	-0.09	4.77	4.77	2.25	2.25	**2.640**	-	-0.129	-0.111
	A-B	B	416	GLN	0.89	0.08	0.07	2.75	2.75	**2.840**	-	0.092	-0.582
	A-B	B	417	ARG	0.28	47.44	44.15	-0.47	-0.47	**2.260**	0.230	0.451	-0.296

Continued on next page

Table 3 – continued from previous page

PDB	Inter	C	Pos	AA	$\Delta\Delta G_{exp}$	ASA^{a}	ASA^{sc}	d^{ad}	d^{m}	FoldX	Robetta	βACV$_{ASA}$	βACV$^{2}_{ASA}$
	A-B	B	418	ASN	0.3	129.65	105.00	-0.17	-0.17	1.610	1.840	0.921	0.929
	A-B	B	419	SER	0.03	25.37	24.84	0.47	0.47	**3.100**	0.980	0.945	1.106
1A4Y	A-B	A	261	TRP	0.1	32.00	32.00	0.35	0.35	1.760	1.060	1.382	-0.069
	A-B	A	263	TRP	1.2	65.96	65.96	-0.29	-0.29	**2.200**	**2.270**	**4.137**	**9.377**
	A-B	A	289	SER	0	7.54	7.54	-0.05	-0.05	1.760	0.600	1.291	0.627
	A-B	A	318	TRP	1.5	42.53	42.53	-0.08	-0.08	**2.440**	**2.190**	1.490	**2.289**
	A-B	A	320	LYS	-0.3	39.47	39.47	0.12	0.12	1.760	-0.210	0.793	1.001
	A-B	A	344	GLU	0.2	16.01	16.01	0.14	0.14	1.790	1.370	0.590	1.937
	A-B	A	375	TRP	1	56.59	56.59	-0.35	-0.35	**2.820**	**2.830**	**2.684**	0.022
	A-B	A	401	GLU	0.9	19.59	19.59	0.44	0.44	1.760	0.020	0.565	0.352
	A-B	A	434	TYR	**3.3**	98.47	98.47	0.10	0.10	**2.850**	**3.000**	**2.386**	**3.957**
	A-B	A	435	ASP	**3.5**	90.55	80.28	-0.10	-0.10	**3.410**	0.570	**3.436**	**3.239**
	A-B	A	437	TYR	0.8	146.14	143.38	0.11	0.11	**4.920**	**3.130**	**2.068**	**2.028**
	A-B	A	457	ARG	-0.2	2.78	2.78	2.62	2.62	1.470	-	-0.664	-0.506
	A-B	A	459	ILE	0.7	23.86	23.86	-0.38	-0.38	**2.270**	0.620	0.937	1.656
	A-B	B	5	ARG	**2.3**	100.13	100.13	-0.43	-0.43	**7.310**	**2.540**	1.712	1.430
	A-B	B	8	HIS	0.9	24.05	24.05	-0.23	-0.23	1.960	0.850	0.882	0.875
	A-B	B	12	GLN	0.3	22.77	22.77	-0.21	-0.21	**2.040**	0.870	**2.100**	1.803
	A-B	B	13	HIS	-0.3	4.70	4.70	1.11	1.11	**2.310**	-	-1.476	-0.737
	A-B	B	31	ARG	0.2	125.12	119.92	-0.58	-0.58	1.760	**2.760**	1.830	1.885
	A-B	B	32	ARG	0.9	64.35	62.16	-0.57	-0.57	**2.660**	0.180	0.103	0.405
	A-B	B	68	ASN	0.2	10.66	10.67	0.64	0.64	1.740	0.520	1.337	1.065
	A-B	B	84	HIS	0.2	63.98	63.97	0.03	0.03	-	1.050	1.030	1.201
	A-B	B	89	TRP	0.2	84.78	84.77	-0.08	-0.08	**4.390**	**2.710**	1.343	1.539
	A-B	B	108	GLU	-0.3	43.90	43.90	0.29	0.29	**3.820**	1.730	1.500	1.502
	A-B	B	114	HIS	0.65	62.62	62.62	0.17	0.33	1.760	1.690	1.155	1.302
1AHW	AB-C	C	156	TYR	**4**	65.88	65.88	-0.22	-0.22	**2.950**	**4.530**	**2.203**	-2.288
	AB-C	C	157	TYR	-1.9	5.75	5.75	1.83	1.83	0.090	-	1.441	-0.417
	AB-C	C	167	THR	0	70.63	41.50	-0.23	-0.20	0.440	-0.240	1.710	0.044
	AB-C	C	170	THR	1	11.52	11.29	0.35	1.83	-0.080	-0.070	0.125	0.915
	AB-C	C	178	ASP	-0.5	14.32	14.32	1.11	1.11	-0.010	-0.090	0.311	0.648
	AB-C	C	197	THR	1.3	22.21	8.87	0.80	1.46	-	-0.020	-0.190	-0.138
	AB-C	C	198	VAL	-0.3	25.99	7.15	-0.35	1.64	-0.010	-0.010	-0.226	-0.087
	AB-C	C	199	ASN	1.1	0.68	0.68	1.33	3.08	-	-	-0.037	-0.003

Continued on next page

Table 3 – continued from previous page

PDB	Inter	C	Pos	AA	$\Delta\Delta G_{exp}$	ASA^{a}	ASA^{sc}	d^{ad}	d^{m}	FoldX	Robetta	βACV$_{ASA}$	βACV$^{2}_{ASA}$
1BRS	A-D	A	27	LYS	**5.4**	56.69	56.69	0.05	0.05	1.590	1.880	**2.947**	**3.827**
	A-D	A	58	ASN	**3.1**	1.85	1.69	0.47	2.28	1.590	-	1.006	1.111
	A-D	A	59	ARG	**5.2**	159.13	147.78	-0.18	-0.18	**2.050**	**3.010**	1.516	1.080
	A-D	A	60	GLU	-0.2	69.39	67.45	-0.21	-0.21	1.590	1.410	1.388	1.203
	A-D	A	73	GLU	**2.8**	10.14	10.14	1.69	1.69	1.590	-	0.934	-0.302
	A-D	A	87	ARG	**5.5**	3.84	3.84	-0.12	-0.12	**3.350**	**4.440**	**2.736**	**3.460**
	A-D	A	102	HIS	**6**	107.74	96.86	-0.27	-0.27	-	**5.080**	**4.587**	**5.036**
	A-D	D	29	TYR	**3.4**	96.78	86.59	-0.15	-0.15	**2.470**	**3.130**	1.899	**3.092**
	A-D	D	35	ASP	**4.5**	115.75	111.62	-0.17	-0.17	1.820	1.420	1.147	1.012
	A-D	D	39	ASP	**7.7**	84.15	84.06	-0.55	-0.55	**6.490**	**9.400**	**3.579**	**4.689**
	A-D	D	42	THR	1.8	39.91	18.77	0.05	0.05	**2.950**	1.660	1.868	1.326
	A-D	D	76	GLU	1.3	24.09	24.10	-0.18	-0.18	**2.670**	1.540	0.588	1.554
1BXI	A-B	A	23	CYS	0.92	38.11	10.61	-0.10	0.08	0.370	-0.080	1.454	1.923
	A-B	A	24	ASN	0.14	20.14	6.41	0.04	1.84	0.060	0.000	0.404	0.313
	A-B	A	27	THR	0.73	12.57	5.33	0.08	0.08	1.390	0.600	1.623	0.850
	A-B	A	29	SER	0.96	9.10	6.56	-0.05	2.87	0.020	-	-0.310	0.242
	A-B	A	30	GLU	1.41	106.09	100.51	-0.35	-0.35	1.350	**2.970**	**2.348**	1.612
	A-B	A	33	LEU	**3.42**	33.40	33.40	0.13	0.13	1.190	1.020	1.068	**2.473**
	A-B	A	34	VAL	**2.58**	61.92	61.50	0.33	0.33	1.220	0.980	0.838	**2.032**
	A-B	A	37	VAL	1.66	16.20	16.20	-0.08	-0.08	0.810	0.500	1.878	**3.018**
	A-B	A	38	THR	0.9	19.24	19.24	0.46	0.46	1.650	1.350	0.986	0.454
	A-B	A	41	GLU	**2.08**	32.84	32.84	-0.30	-0.30	0.730	-0.080	**3.708**	**3.903**
	A-B	A	48	SER	0.01	9.38	2.14	-0.05	3.00	0.360	-	0.341	0.257
	A-B	A	50	SER	**2.19**	35.71	33.03	-0.25	-0.25	0.890	**5.400**	1.338	0.985
	A-B	A	51	ASP	**5.92**	36.70	36.63	-0.39	-0.39	**2.160**	0.820	1.701	0.973
	A-B	A	53	ILE	0.85	12.73	2.21	-0.49	-0.15	0.750	0.170	**2.218**	0.953
	A-B	A	54	TYR	**4.83**	98.94	80.73	-0.25	-0.25	**3.250**	**2.860**	1.943	**4.323**
	A-B	A	55	TYR	**4.63**	90.64	90.63	-0.25	-0.25	**3.600**	**3.490**	1.826	0.651
	A-B	A	56	PRO	1.24	5.40	5.41	0.53	0.53	1.410	-	1.256	1.589
1CBW	GH-I	I	11	THR	0.22	39.50	39.50	0.08	0.08	0.550	0.180	0.272	-0.307
	GH-I	I	13	PRO	-0.06	59.11	47.88	-0.10	0.62	0.610	-	0.373	0.308
	GH-I	I	15	LYS	1.99	176.99	143.34	-0.65	-0.19	0.190	1.540	**3.070**	**3.960**
	GH-I	I	17	ARG	0.55	171.37	146.39	-0.42	-0.42	-0.010	1.820	1.267	1.501
	GH-I	I	18	ILE	1.40	28.33	28.33	1.04	1.17	1.100	0.730	0.603	1.308

Continued on next page

Table 3 – continued from previous page

PDB	Inter	C	Pos	AA	$\Delta\Delta G_{exp}$	ASA^a	ASA^{sc}	d^{ad}	d^m	FoldX	Robetta	βACV$_{ASA}$	βACV$^2_{ASA}$
	GH-I	I	19	ILE	0.14	28.17	27.90	-0.23	-0.23	0.730	0.540	-0.943	-0.903
	GH-I	I	34	VAL	0.05	8.69	8.69	1.06	1.06	0.450	0.260	0.909	0.176
	GH-I	I	39	ARG	0.22	67.80	62.57	0.31	0.31	0.490	1.330	0.120	-0.169
1DAN	HL-TU	H	129B	ARG	0	1.34	1.33	2.63	2.63	-	-	-0.619	-0.867
	HL-TU	H	129F	PHE	0	96.60	71.15	0.01	0.01	-	1.660	**2.372**	-0.729
	HL-TU	H	134	ARG	0.51	126.88	120.63	-0.20	-0.20	-0.770	**3.090**	1.891	1.662
	HL-TU	H	135	PHE	0	26.38	25.83	0.59	0.59	0.380	0.530	0.597	0.573
	HL-TU	H	162	ARG	0.65	4.26	2.85	1.74	1.74	0.340	-	1.711	**2.990**
	HL-TU	H	164	MET	0.5	111.85	110.80	-0.17	-0.17	1.160	1.030	1.552	0.975
	HL-TU	H	165	THR	0	8.75	6.03	-0.03	0.85	0.300	0.270	0.723	1.041
	HL-TU	H	166	GLN	0	73.86	72.81	-0.14	-0.03	1.230	**2.600**	1.071	0.733
	HL-TU	H	167	ASP	0.41	42.46	42.46	-0.12	-0.12	1.290	1.340	**2.204**	1.176
	HL-TU	H	170A	GLN	0	0.00	0.00	2.76	2.76	-	-	0.014	0.270
	HL-TU	H	170	GLN	0	4.96	4.96	1.66	1.66	0.670	-	0.452	0.740
	HL-TU	H	230	ARG	0.51	30.87	30.87	-0.01	-0.01	1.180	1.330	0.186	-0.572
	HL-TU	L	39	LEU	0	107.16	103.32	0.01	0.01	0.960	1.240	0.464	-0.288
	HL-TU	L	42	ILE	0	3.54	3.53	2.00	2.00	0.250	0.080	0.212	0.748
	HL-TU	L	62	LYS	0	71.99	71.99	-0.25	-0.25	-0.010	-0.270	0.677	0.905
	HL-TU	L	64	GLN	0.8	62.34	60.52	-0.10	-0.10	0.250	**2.980**	1.158	0.636
	HL-TU	L	69	ILE	1.9	74.69	74.69	-0.15	-0.15	1.140	1.570	1.510	**2.549**
	HL-TU	L	71	PHE	1.2	107.57	107.49	-0.49	-0.49	**2.370**	**3.070**	1.170	0.392
	HL-TU	L	73	LEU	0	1.04	0.00	-0.17	2.98	1.050	-	0.162	0.190
	HL-TU	L	77	GLU	0	60.26	60.26	0.09	0.09	0.280	0.360	1.236	1.591
	HL-TU	L	79	ARG	1.2	119.07	110.44	-0.31	-0.31	**2.060**	**2.450**	-0.388	-0.227
	HL-TU	L	88	GLN	0	29.41	29.41	0.29	0.29	1.020	0.350	0.102	-0.092
	HL-TU	L	92	VAL	0	79.29	65.59	-0.10	-0.10	1.990	1.050	0.625	0.332
	HL-TU	L	93	ASN	0	29.29	29.28	-0.24	-0.10	1.360	0.120	0.867	0.368
	HL-TU	L	94	GLU	0	39.69	38.31	0.51	0.51	0.800	-	0.149	0.258
	HL-TU	T	15	LYS	-0.4	3.32	3.32	2.23	2.29	0.970	-	-0.420	-0.158
	HL-TU	T	17	THR	0.1	28.20	25.69	-0.15	-0.15	1.130	0.130	0.372	0.250
	HL-TU	T	18	ASN	0.2	11.27	11.27	0.50	0.97	1.090	0.040	0.916	1.048
	HL-TU	T	20	LYS	**2.6**	75.90	75.89	0.05	0.05	**2.810**	1.500	1.714	**2.736**
	HL-TU	T	22	ILE	0.7	27.72	27.72	0.24	0.24	**2.640**	0.650	1.182	-0.595
	HL-TU	T	24	GLU	0.7	14.75	14.74	-0.31	-0.31	**2.230**	0.640	0.007	0.407

Continued on next page

Table 3 – continued from previous page

PDB	Inter	C	Pos	AA	$\Delta\Delta G_{exp}$	ASA^{a}	ASA^{sc}	d^{ad}	d^{m}	FoldX	Robetta	βACV$_{ASA}$	βACV$^{2}_{ASA}$
	HL-TU	T	37	GLN	0.55	27.30	27.30	0.14	0.14	**3.370**	1.410	1.758	**2.220**
	HL-TU	T	41	LYS	0.35	22.37	3.21	0.39	2.87	1.420	-	-0.139	0.087
	HL-TU	T	42	SER	-0.1	35.00	18.10	0.34	2.40	1.420	-0.050	-0.114	-0.040
	HL-TU	T	44	ASP	0.7	51.16	51.16	-0.18	-0.18	**2.520**	0.890	1.016	1.071
	HL-TU	T	45	TRP	1.6	61.09	49.10	0.08	0.20	1.420	1.050	1.355	$\overline{\wedge}$
	HL-TU	T	46	LYS	0.25	39.11	39.11	0.51	0.51	1.550	-	0.574	0.594
	HL-TU	T	47	SER	0.05	26.81	22.94	0.31	0.83	1.160	0.600	0.842	0.407
	HL-TU	T	48	LYS	0.4	22.83	22.83	0.09	0.09	1.840	0.430	1.018	1.442
	HL-TU	T	50	PHE	0.4	101.62	101.18	-0.24	-0.24	**4.060**	**2.610**	1.229	-0.395
	HL-TU	T	51	TYR	-0.1	34.10	34.11	0.43	0.43	**2.110**	-	-0.365	0.523
	HL-TU	T	58	ASP	**2.18**	48.82	43.19	-0.24	-0.23	**2.560**	1.090	**3.011**	**2.302**
	HL-TU	T	61	ASP	0.24	50.59	50.26	-0.16	-0.16	0.640	0.010	0.713	0.688
	HL-TU	T	62	GLU	0	1.07	1.08	2.38	2.38	1.260	-	1.263	1.866
	HL-TU	T	72	LEU	-0.06	0.24	0.24	2.65	3.11	1.430	-	0.591	0.148
	HL-TU	T	76	PHE	1.2	15.98	15.98	0.22	0.22	1.700	0.620	**2.068**	1.469
	HL-TU	T	78	TYR	0.7	0.26	0.26	2.78	2.78	1.400	-	-0.133	1.281
	HL-TU	U	94	TYR	1	91.98	76.28	-0.14	-0.14	**2.140**	**2.700**	0.709	1.709
	HL-TU	U	110	GLN	1.4	69.94	62.32	-0.17	-0.17	0.750	1.690	0.675	0.209
	HL-TU	U	128	GLU	0.1	14.49	14.49	0.79	0.79	0.750	-0.110	0.148	0.358
	HL-TU	U	131	ARG	0	66.22	59.92	-0.49	-0.49	0.750	0.280	0.438	-0.091
	HL-TU	U	132	THR	0	1.04	0.00	0.25	3.05	0.750	-	0.069	-0.337
	HL-TU	U	133	LEU	0	41.56	41.56	-0.05	-0.05	1.320	1.620	**2.187**	**5.061**
	HL-TU	U	135	ARG	0.55	69.95	69.96	-0.48	-0.48	0.750	0.940	0.992	1.791
	HL-TU	U	138	ASN	0	18.61	15.84	1.61	1.61	0.750	-	-0.306	-0.061
	HL-TU	U	140	PHE	1.5	23.34	23.33	0.42	0.42	0.910	1.540	1.072	1.393
	HL-TU	U	163	SER	0	28.06	24.60	-0.36	-0.36	0.750	0.420	0.232	0.005
	HL-TU	U	203	THR	0.1	25.60	25.61	0.19	0.19	0.750	0.220	0.757	0.594
	HL-TU	U	207	VAL	-0.2	73.65	54.98	0.02	0.02	1.960	1.120	1.122	-0.554
	HL-TU	U	208	GLU	0	25.82	25.82	0.33	0.33	0.750	0.280	0.111	0.376
1DFJ	E-I	I	202	GLU	1	24.08	24.08	0.14	0.14	-0.480	0.770	1.388	0.072
	E-I	I	257	TRP	1.3	39.18	39.18	0.14	0.14	1.030	1.570	**2.607**	0.254
	E-I	I	259	TRP	**2.2**	43.45	43.45	-0.02	-0.02	0.910	**3.090**	**2.788**	**2.373**
	E-I	I	283	GLU	1.3	23.90	23.90	0.01	0.01	0.290	-0.100	-0.307	-0.188
	E-I	I	285	SER	0.8	2.18	2.18	1.72	1.72	0.410	0.000	0.704	-0.048

Continued on next page

Table 3 – continued from previous page

PDB	Inter	C	Pos	AA	$\Delta\Delta G_{exp}$	ASA^a	ASA^{sc}	d^{ad}	d^m	FoldX	Robetta	βACV$_{ASA}$	βACV$^2_{ASA}$
	E-I	I	314	TRP	1	54.72	54.72	0.22	0.22	1.150	1.270	0.857	1.983
	E-I	I	316	LYS	1.3	42.17	42.17	0.32	0.32	-0.150	0.380	0.355	0.570
	E-I	I	397	GLU	1.3	26.34	26.34	-0.31	-0.31	-0.100	0.420	0.509	0.509
	E-I	I	430	TYR	**5.9**	107.72	107.72	-0.11	-0.11	**3.750**	**2.720**	**2.247**	**5.802**
	E-I	I	431	ASP	**3.6**	77.71	62.73	0.36	0.36	0.090	0.160	-0.126	0.121
	E-I	I	433	TYR	**2.6**	163.26	158.27	-0.34	-0.34	**3.190**	**3.620**	**2.237**	1.940
	E-I	I	453	ARG	0.8	28.63	28.62	-0.23	-0.23	0.810	0.210	-0.208	-0.224
	E-I	I	455	ILE	0.3	35.29	35.29	0.11	0.11	**2.010**	0.500	1.189	1.254
1DVF	AB-CD	A	30	HIS	1.7	18.00	18.00	0.35	0.35	0.140	0.260	0.277	0.426
	AB-CD	A	32	TYR	**2**	44.27	44.28	0.16	0.16	0.060	0.430	**2.385**	1.193
	AB-CD	A	49	TYR	1.7	23.07	23.07	-0.23	-0.23	1.040	0.870	1.476	0.670
	AB-CD	A	50	TYR	0.7	53.01	53.01	-0.12	-0.12	0.060	0.550	1.014	**2.055**
	AB-CD	A	92	TRP	0.3	74.62	55.78	-0.20	0.01	0.470	1.550	1.834	0.216
	AB-CD	A	93	SER	1.2	11.38	11.38	0.99	3.22	0.130	-0.020	-0.173	-0.304
	AB-CD	B	30	THR	0.9	38.44	18.03	-0.12	1.32	-0.080	0.010	0.232	0.244
	AB-CD	B	32	TYR	1.8	24.36	22.14	0.16	1.29	0.840	0.120	1.184	0.853
	AB-CD	B	52	TRP	**4.2**	65.45	65.46	-0.13	-0.13	**2.390**	**3.260**	**3.575**	1.540
	AB-CD	B	54	ASP	**4.3**	68.05	65.23	-0.24	-0.24	**2.130**	0.480	1.953	**2.479**
	AB-CD	B	56	ASN	1.2	53.22	53.22	-0.29	-0.15	0.890	1.060	0.735	1.287
	AB-CD	B	58	ASP	1.6	14.14	14.14	0.14	0.14	1.270	0.710	1.012	1.234
	AB-CD	B	98	GLU	**4.2**	10.64	7.42	-0.26	-0.26	**2.930**	1.150	**5.462**	**4.141**
	AB-CD	B	99	ARG	1.9	59.96	59.96	0.16	0.16	-0.160	0.220	0.526	0.892
	AB-CD	B	100	ASP	**2.8**	56.32	46.95	0.00	0.00	-0.340	0.430	1.122	0.930
	AB-CD	B	101	TYR	**4**	69.34	67.93	-0.04	-0.04	1.840	**2.190**	**4.101**	**5.855**
	AB-CD	C	49	TYR	1.9	15.95	15.95	-0.24	-0.24	1.640	0.040	0.479	0.530
	AB-CD	D	33	HIS	1.9	26.99	26.99	0.00	0.00	0.650	0.790	-0.203	-0.131
	AB-CD	D	52	ASP	1.7	15.78	15.78	0.48	0.48	0.230	-0.230	1.005	0.034
	AB-CD	D	54	ASN	1.9	46.18	46.18	-0.23	-0.23	1.140	**2.090**	1.004	0.917
	AB-CD	D	97	ILE	**2.7**	49.54	49.55	-0.10	-0.10	1.900	1.070	0.715	0.182
	AB-CD	D	98	TYR	**4.7**	188.42	165.71	-0.26	-0.26	**5.780**	**5.710**	**2.351**	**4.645**
	AB-CD	D	100B	ARG	**4.1**	92.07	92.07	-0.20	-0.20	-	1.660	**2.809**	**2.605**
	AB-CD	D	100	GLN	1.6	96.01	90.58	-0.29	-0.29	1.540	1.440	0.959	0.730
1DX5	I-M	M	34	PHE	**2.6**	15.87	15.87	0.32	0.32	**3.400**	1.010	1.088	0.981
	I-M	M	36A	SER	-0.2	17.80	17.79	-0.05	-0.05	-	0.570	0.477	0.523

Continued on next page

Table 3 – continued from previous page

PDB	Inter	C	Pos	AA	$\Delta\Delta G_{exp}$	ASA^a	ASA^{sc}	d^{ad}	d^m	FoldX	Robetta	βACV$_{ASA}$	βACV$^2_{ASA}$
	I-M	M	37	PRO	1.2	53.02	53.02	0.12	0.73	**2.410**	-	0.315	0.462
	I-M	M	38	GLN	1.4	116.04	92.86	-0.24	-0.23	**2.870**	**2.310**	1.804	1.454
	I-M	M	39	GLU	-0.2	6.82	6.82	2.00	2.39	1.610	-0.090	0.511	0.622
	I-M	M	65	LEU	1	21.78	21.78	0.49	0.49	**2.060**	-	0.890	1.725
	I-M	M	67	ARG	**3.4**	14.96	14.96	-0.30	-0.30	**3.220**	-0.010	**6.244**	**6.003**
	I-M	M	74	THR	0.8	42.62	22.45	-0.23	-0.23	1.610	0.200	0.272	0.236
	I-M	M	75	ARG	0.7	32.74	32.74	0.05	0.13	1.900	0.050	0.044	0.478
	I-M	M	76	TYR	**3**	87.26	81.60	-0.23	-0.15	**3.790**	**2.030**	**2.617**	**3.018**
	I-M	M	77A	ARG	1.5	86.70	86.70	-0.25	-0.25	-	**2.070**	1.006	0.365
	I-M	M	80	GLU	**3.4**	2.26	0.00	1.52	1.52	1.610	-	**3.078**	**3.294**
	I-M	M	81	LYS	1	44.74	44.74	0.32	0.42	**2.890**	0.800	1.105	1.600
	I-M	M	82	ILE	**2.6**	71.53	47.74	-0.22	0.15	**3.690**	1.070	1.848	**2.790**
	I-M	M	84	MET	0.3	54.10	54.10	-0.14	-0.14	**3.060**	0.770	0.498	0.898
	I-M	M	110	LYS	0	52.26	52.27	0.18	0.18	1.910	0.170	0.434	0.544
1F47	A-B	A	4	ASP	0.7	56.93	32.43	-0.26	0.07	0.110	-0.150	0.508	0.877
	A-B	A	5	TYR	0.88	105.84	96.36	-0.31	-0.31	0.620	1.650	0.820	1.050
	A-B	A	6	LEU	0.94	96.95	89.61	-0.21	-0.01	1.440	1.940	1.360	**2.096**
	A-B	A	7	ASP	1.76	15.11	12.44	0.91	1.82	0.280	-	-0.340	-0.610
	A-B	A	8	ILE	**2.56**	85.67	85.67	0.08	0.08	**2.760**	**2.070**	1.129	**2.451**
	A-B	A	9	PRO	-0.06	4.74	4.74	1.80	1.80	0.230	-	0.222	-0.031
	A-B	A	11	PHE	**2.49**	91.36	90.43	-0.27	-0.27	**3.180**	**2.430**	1.813	**2.142**
	A-B	A	12	LEU	**2.33**	67.29	67.29	0.08	0.08	1.650	1.180	1.108	1.623
	A-B	A	15	GLN	-0.05	55.53	55.21	-0.80	-0.56	-0.340	0.040	0.828	0.683
1FC2	C-D	C	147	ASN	0.6	47.72	46.00	-0.30	-0.30	0.880	0.290	0.483	0.468
	C-D	C	150	ILE	**2.2**	26.04	25.51	0.27	0.27	0.450	0.810	1.519	0.642
	C-D	C	154	LYS	1.2	59.74	59.74	0.12	0.12	-0.310	0.180	1.043	0.594
1FCC	A-C	C	25	THR	0.24	27.59	27.59	1.10	1.10	-0.020	0.100	0.664	0.581
	A-C	C	27	GLU	**4.9**	59.61	59.61	-0.56	-0.56	**4.060**	**3.140**	**3.872**	**3.590**
	A-C	C	28	LYS	1.3	141.49	134.63	-0.21	-0.21	0.390	0.990	1.451	1.782
	A-C	C	31	LYS	**3.5**	73.61	72.13	-0.21	-0.21	0.290	1.910	**3.865**	**4.237**
	A-C	C	35	ASN	**2.4**	80.44	80.44	-0.61	-0.61	-1.620	1.180	**2.048**	**2.426**
	A-C	C	40	ASP	0.3	41.07	41.06	0.10	0.10	-0.310	-0.150	0.294	0.797
	A-C	C	42	GLU	0.4	38.85	38.85	-0.37	-0.37	-0.490	0.030	0.141	0.725
	A-C	C	43	TRP	**3.8**	55.53	30.91	-0.26	-0.24	1.250	**2.710**	**2.033**	**8.300**

Continued on next page

Table 3 – continued from previous page

PDB	Inter	C	Pos	AA	$\Delta\Delta G_{exp}$	ASA^{a}	ASA^{sc}	d^{ad}	d^{m}	FoldX	Robetta	βACV$_{ASA}$	βACV$^{2}_{ASA}$
1GC1	C-G	C	23	SER	0.29	4.22	4.22	1.42	1.42	0.290	-	0.509	0.656
	C-G	C	25	GLN	0.03	20.08	20.08	-0.01	-0.01	0.030	0.420	0.176	0.942
	C-G	C	27	HIS	0.28	38.62	38.62	-0.39	-0.39	1.010	0.800	0.323	0.527
	C-G	C	29	LYS	0.59	24.95	24.95	-0.24	-0.24	**2.430**	**2.460**	1.462	1.484
	C-G	C	32	ASN	0.18	15.44	3.01	-0.18	2.44	0.540	0.000	0.211	0.233
	C-G	C	33	GLN	0.1	28.86	17.33	-0.34	1.38	0.690	0.030	0.703	0.726
	C-G	C	35	LYS	0.32	84.45	83.89	-0.28	-0.28	-0.210	0.550	1.459	0.968
	C-G	C	40	GLN	-0.41	87.86	78.97	-0.16	0.03	-0.700	1.660	0.622	0.138
	C-G	C	42	SER	0	75.02	67.54	-0.14	-0.12	-0.800	0.030	1.237	0.393
	C-G	C	44	LEU	1.04	29.13	7.83	0.23	1.40	0.690	0.070	0.463	-0.835
	C-G	C	45	THR	-0.15	23.68	23.69	0.05	0.05	0.750	0.320	1.273	1.314
	C-G	C	52	ASN	0.7	23.31	23.31	-0.08	-0.08	0.640	1.010	1.349	1.325
	C-G	C	59	ARG	1.16	56.12	47.19	-0.57	-0.57	1.440	1.020	1.302	0.237
	C-G	C	60	SER	-0.09	54.63	39.70	-0.38	1.26	-	0.140	0.476	0.254
	C-G	C	63	ASP	-0.32	36.61	36.61	1.05	1.05	0.070	-0.050	0.719	0.582
	C-G	C	64	GLN	0.44	29.57	29.56	0.31	0.31	0.680	1.020	0.267	0.402
	C-G	C	85	GLU	1.31	1.77	1.77	1.30	1.30	0.770	-	-0.114	0.415
1JCK	A-B	B	20	THR	1.4	59.98	59.68	0.03	0.03	-0.370	1.260	1.075	0.317
	A-B	B	23	ASN	**2.5**	49.05	49.05	-0.38	-0.38	-1.120	1.960	**2.483**	**2.346**
	A-B	B	26	TYR	1.7	38.74	38.73	-0.33	-0.33	0.260	0.920	1.604	1.888
	A-B	B	60	ASN	1.3	42.33	42.33	0.29	0.29	-0.370	0.820	1.160	0.691
	A-B	B	90	TYR	**2.5**	32.16	31.73	-0.17	-0.13	0.710	1.000	1.012	⊼
	A-B	B	91	VAL	**2.1**	71.86	71.68	-0.16	-0.16	0.220	1.040	0.590	-2.089
	A-B	B	103	LYS	0.4	35.30	19.12	-0.54	1.44	-0.170	-0.260	**2.121**	**2.663**
	A-B	B	176	PHE	1.9	44.52	44.52	0.27	0.27	-0.270	0.630	-0.126	0.627
	A-B	B	210	GLN	**2.5**	14.05	14.05	-0.30	-0.30	-0.140	1.120	1.550	**2.431**
1JRH	HL-I	H	32	TYR	1.4	60.47	60.48	0.10	0.10	**2.090**	1.630	0.874	1.295
	HL-I	H	52	TRP	**2.7**	18.74	18.74	0.17	0.17	1.310	1.550	**5.463**	**3.700**
	HL-I	H	53	TRP	**2.4**	27.95	27.96	-0.13	-0.13	1.730	0.730	1.713	**3.145**
	HL-I	H	54	ASP	1.9	11.50	11.50	-0.08	-0.08	0.750	1.640	0.577	0.975
	HL-I	H	56	ASP	1.8	18.11	18.11	-0.08	-0.08	0.420	0.490	1.178	1.044
	HL-I	H	58	TYR	1.2	48.16	48.16	0.18	0.18	0.940	**2.050**	1.664	1.114
	HL-I	H	95	ARG	0.54	12.49	12.49	-0.12	-0.12	1.850	1.480	-0.036	-0.271
	HL-I	H	99	TYR	1.1	87.97	87.98	-0.35	-0.35	1.610	1.980	1.023	1.871

Continued on next page

Table 3 – continued from previous page

PDB	Inter	C	Pos	AA	$\Delta\Delta G_{exp}$	ASA^{a}	ASA^{sc}	d^{ad}	d^{m}	FoldX	Robetta	βACV$_{ASA}$	βACV$^{2}_{ASA}$
	HL-I	H	100B	HIS	1.7	49.52	49.52	-0.13	-0.13	-	**3.380**	1.685	**2.977**
	HL-I	I	47	LYS	**3.6**	36.96	36.96	-0.08	-0.08	**2.550**	1.400	**4.665**	**4.751**
	HL-I	I	48	ASN	-0.3	0.00	0.00	2.16	2.16	0.280	-	0.729	0.605
	HL-I	I	49	TYR	**3.4**	113.37	112.48	-0.26	-0.26	**3.520**	**3.870**	**3.959**	**3.799**
	HL-I	I	51	VAL	1.9	93.80	68.43	-0.13	-0.13	1.750	1.000	1.570	0.505
	HL-I	I	52	LYS	**3**	105.93	104.00	-0.08	-0.08	1.530	**2.380**	1.228	0.654
	HL-I	I	53	ASN	**3.9**	114.90	95.90	-0.17	-0.17	0.810	**2.720**	**2.246**	**3.766**
	HL-I	I	54	SER	0.3	21.04	14.86	0.19	2.50	0.180	-0.030	0.286	0.415
	HL-I	I	55	GLU	-0.4	60.34	60.28	-0.23	0.08	0.250	-0.100	-0.320	-0.618
	HL-I	I	79	ASN	-0.4	0.00	0.00	0.63	1.99	0.230	-	1.887	1.956
	HL-I	I	82	TRP	**4.5**	45.68	45.68	0.25	0.25	1.740	1.350	1.087	1.247
	HL-I	I	84	ARG	-0.3	19.08	19.08	0.60	0.60	0.650	0.370	0.217	0.600
	HL-I	I	98	LYS	0	64.43	64.43	-0.12	-0.12	-0.260	0.050	0.349	0.245
	HL-I	L	27	GLU	0.54	39.52	39.52	0.48	0.48	0.040	0.670	0.588	0.549
	HL-I	L	28	ASP	0.44	27.51	23.86	-0.12	0.55	0.540	0.670	0.770	0.356
	HL-I	L	30	TYR	1.1	67.76	67.76	-0.10	-0.10	0.960	1.090	1.685	1.132
	HL-I	L	91	TYR	0.58	5.32	0.06	0.18	0.28	0.260	0.280	1.072	1.659
	HL-I	L	92	TRP	**2.8**	114.91	90.24	-0.08	-0.03	**2.790**	**3.010**	**3.398**	**3.989**
	HL-I	L	93	SER	-0.65	46.74	46.72	-0.23	-0.23	-0.190	0.540	0.625	0.347
	HL-I	L	94	THR	0.38	37.77	31.33	-0.17	0.08	0.230	0.360	0.715	0.563
	HL-I	L	96	TRP	1.7	2.98	2.99	0.26	0.26	0.470	0.590	**4.821**	1.387
1JTG	A-B	A	104	GLU	1.55	98.59	94.89	-0.22	-0.22	**3.650**	**2.100**	1.752	-0.953
	A-B	A	105	TYR	-0.17	154.63	145.98	-0.27	-0.27	**3.930**	**4.490**	**2.988**	**2.601**
	A-B	A	130	SER	0.33	21.34	20.74	-0.23	-0.23	**3.020**	-0.180	-0.208	-1.082
	A-B	A	234	LYS	1	0.00	0.00	0.23	0.23	**4.270**	0.600	**4.279**	**4.501**
	A-B	A	235	SER	1.3	4.21	3.95	-0.01	-0.01	**3.820**	**2.330**	0.678	-0.629
	A-B	A	243	ARG	1.27	13.40	13.40	-0.30	-0.30	**3.960**	**3.760**	**3.934**	**3.485**
	A-B	B	49	ASP	1.8	138.08	115.15	-0.30	-0.30	**8.530**	**6.420**	**2.355**	**3.392**
	A-B	B	74	LYS	**3.56**	26.41	26.41	-0.27	-0.27	**6.420**	0.680	**6.571**	**5.091**
	A-B	B	142	PHE	**2.1**	142.58	135.37	-0.06	-0.01	**5.350**	**3.230**	**2.122**	**3.348**
	A-B	B	143	TYR	0.38	28.88	23.82	-0.11	-0.09	**4.810**	0.830	0.866	$\veebar$
1NMB	HL-N	H	99	TYR	1.5	59.68	59.69	-0.01	-0.01	0.320	1.280	-0.238	0.821
1VFB	AB-C	A	30	HIS	0.8	28.66	28.67	0.33	0.33	0.400	0.260	0.486	0.684
	AB-C	A	32	TYR	1.3	54.48	54.49	0.14	0.14	**2.630**	1.310	**2.206**	1.466

Continued on next page

Table 3 – continued from previous page

PDB	Inter	C	Pos	AA	$\Delta\Delta G_{exp}$	ASA^a	ASA^{sc}	d^{ad}	d^m	FoldX	Robetta	βACV$_{ASA}$	βACV$^2_{ASA}$
	AB-C	A	49	TYR	0.8	21.06	21.06	0.38	0.38	0.610	0.490	1.022	1.417
	AB-C	A	50	TYR	0.4	66.18	66.18	-0.06	-0.06	**2.610**	1.520	1.760	**4.406**
	AB-C	A	53	THR	-0.23	17.71	17.71	-0.25	-0.25	1.170	0.640	0.538	0.558
	AB-C	A	92	TRP	1.71	60.86	60.86	0.15	0.15	**3.290**	**2.060**	1.965	-1.066
	AB-C	A	93	SER	0.11	42.22	35.45	-0.16	0.63	0.050	0.000	0.487	0.672
	AB-C	B	32	TYR	0.5	30.94	30.52	0.37	0.37	0.510	0.660	0.917	**2.080**
	AB-C	B	52	TRP	1.23	53.78	53.78	-0.12	-0.12	1.520	1.750	**2.530**	**2.592**
	AB-C	B	54	ASP	1.95	36.05	32.45	0.29	0.29	-0.500	-0.140	0.598	1.140
	AB-C	B	58	ASP	-0.2	0.00	0.00	2.98	2.98	0.040	-	0.540	0.887
	AB-C	B	99	ARG	0.47	48.98	48.98	-0.37	-0.37	-0.100	0.730	1.640	**2.829**
	AB-C	B	100	ASP	**3.1**	61.62	60.34	-0.14	-0.14	**2.600**	**3.030**	**3.747**	**4.773**
	AB-C	B	101	TYR	**4**	68.40	68.40	-0.13	-0.13	**4.890**	**3.230**	**2.997**	**6.263**
	AB-C	C	18	ASP	0.3	32.14	31.89	-0.06	-0.06	**2.310**	0.550	0.704	0.406
	AB-C	C	19	ASN	0.3	69.81	69.81	-0.25	-0.25	1.130	1.000	1.924	1.396
	AB-C	C	23	TYR	0.4	9.27	9.27	0.23	1.41	0.660	-	0.008	$\veebar$
	AB-C	C	24	SER	0.8	43.53	41.63	-0.14	0.04	0.540	0.710	1.088	0.866
	AB-C	C	116	LYS	0.7	61.49	52.60	0.31	0.31	0.680	0.850	1.225	0.575
	AB-C	C	118	THR	0.8	44.15	33.90	0.04	0.29	0.900	0.100	0.567	0.816
	AB-C	C	119	ASP	1	44.87	40.94	-0.12	-0.10	1.570	1.670	1.505	1.386
	AB-C	C	120	VAL	0.9	13.75	13.27	0.12	0.12	1.370	0.230	1.630	**6.105**
	AB-C	C	121	GLN	**2.9**	120.40	119.81	-0.23	-0.23	**5.330**	**4.200**	**3.120**	**2.683**
	AB-C	C	124	ILE	1.2	22.89	21.77	0.49	0.49	1.580	-	1.203	1.759
	AB-C	C	125	ARG	1.8	56.47	56.47	0.12	0.12	**2.450**	**2.220**	0.608	1.174
	AB-C	C	129	LEU	0.2	28.38	0.00	0.33	0.33	0.840	-	0.081	$\veebar$
2PTC	E-I	I	15	LYS	**10.05**	201.11	165.20	-0.48	0.06	1.560	**4.160**	**4.115**	**2.994**
	E-I	I	18	ILE	**4.97**	56.56	56.56	0.54	0.54	1.510	0.940	1.260	**2.177**
3HFM	HL-Y	H	31	SER	0.18	40.97	40.12	-0.59	-0.59	-0.140	0.380	0.795	0.572
	HL-Y	H	32	ASP	1.93	36.26	31.97	0.49	0.71	0.610	1.100	0.462	0.833
	HL-Y	H	33	TYR	**6**	74.92	74.92	-0.05	-0.05	**2.770**	**2.900**	**3.916**	**7.609**
	HL-Y	H	50	TYR	**7.4**	16.75	16.75	-0.54	-0.54	0.460	**2.960**	**5.108**	**3.166**
	HL-Y	H	53	TYR	**3.3**	91.75	89.66	-0.49	-0.26	1.510	1.660	**2.160**	**4.738**
	HL-Y	H	58	TYR	1.7	45.89	45.89	-0.50	-0.50	-0.630	1.770	1.195	**3.219**
	HL-Y	H	98	TRP	**5.5**	52.02	52.02	0.12	0.12	0.870	0.990	**3.252**	**4.288**
	HL-Y	L	31	ASN	**5.2**	53.35	53.35	-0.20	-0.16	1.450	1.860	1.159	1.470

Continued on next page

Table 3 – continued from previous page

PDB	Inter	C	Pos	AA	$\Delta\Delta G_{exp}$	ASA^a	ASA^{sc}	d^{ad}	d^m	FoldX	Robetta	βACV$_{ASA}$	βACV$^2_{ASA}$
	HL-Y	L	32	ASN	**5.13**	29.83	29.83	0.19	0.19	1.320	**2.480**	**3.055**	**3.477**
	HL-Y	L	50	TYR	**4.6**	57.84	57.84	-0.39	-0.39	1.090	1.400	**2.913**	-1.497
	HL-Y	L	53	GLN	0.96	53.23	53.23	-0.45	-0.45	1.640	0.830	1.079	0.828
	HL-Y	L	96	TYR	**2.73**	16.30	16.31	-0.16	-0.16	-0.730	0.450	**3.018**	**2.763**
	HL-Y	Y	15	HIS	-0.44	16.36	12.91	-0.09	0.98	-	0.100	-0.195	-0.039
	HL-Y	Y	20	TYR	**4.9**	66.59	66.59	0.02	0.02	0.800	**2.230**	**3.631**	**8.701**
	HL-Y	Y	21	ARG	1.07	121.93	118.40	-0.54	-0.54	-0.790	**3.420**	**2.229**	1.363
	HL-Y	Y	63	TRP	0.31	22.90	22.90	-0.17	-0.17	1.190	0.830	1.938	0.828
	HL-Y	Y	73	ARG	-0.33	67.88	67.88	-0.60	-0.60	-0.460	0.620	0.886	0.594
	HL-Y	Y	75	LEU	0.69	61.94	58.98	-0.06	-0.06	1.140	1.310	1.829	1.060
	HL-Y	Y	89	THR	0	15.37	15.37	0.36	0.36	0.300	0.170	0.009	0.268
	HL-Y	Y	93	ASN	0.21	48.27	44.04	-0.45	-0.45	1.650	1.530	1.045	0.988
	HL-Y	Y	96	LYS	**7**	53.30	51.52	-0.39	-0.39	1.200	**2.130**	**3.384**	**4.369**
	HL-Y	Y	97	LYS	**6.2**	77.64	72.16	-0.05	0.14	0.910	1.420	1.359	1.244
	HL-Y	Y	98	ILE	0	2.53	2.10	0.20	1.59	0.080	-	0.843	0.531
	HL-Y	Y	100	SER	0.26	41.61	23.24	0.01	0.16	-0.150	0.840	**2.056**	1.886
	HL-Y	Y	101	ASP	0.94	93.94	84.66	-0.49	-0.49	0.070	0.320	**2.788**	**2.799**
3HHR	A-BC	A	2	PRO	-0.05	86.40	75.92	0.09	0.09	1.420	-	0.160	0.135
	A-BC	A	3	THR	-0.05	4.59	0.77	1.72	3.16	-	0.010	-0.211	-0.476
	A-BC	A	4	ILE	0.41	69.54	68.36	0.40	0.40	0.330	1.050	0.699	0.103
	A-BC	A	5	PRO	0.43	0.00	0.00	2.86	2.86	-	-	0.255	0.400
	A-BC	A	8	ARG	0.2	88.45	88.45	0.14	0.14	-2.140	1.360	1.030	1.384
	A-BC	A	9	LEU	-0.04	18.77	14.72	-0.02	-0.02	0.320	0.440	1.031	1.934
	A-BC	A	12	ASN	0.1	59.83	59.14	-0.33	-0.33	0.020	**3.020**	**2.881**	1.872
	A-BC	A	15	LEU	0.15	75.00	70.45	-0.19	0.18	0.280	1.260	1.429	0.918
	A-BC	A	16	ARG	0.24	80.77	80.77	-0.21	-0.21	0.960	**2.780**	**3.592**	**4.375**
	A-BC	A	19	ARG	0.05	44.37	44.37	0.74	1.02	0.120	0.230	-0.059	-0.670
	A-BC	B	108	CYS	0	9.97	9.97	0.47	0.47	0.130	-0.040	1.002	0.941
	A-BC	B	122	CYS	0	71.60	37.92	-0.09	1.08	0.210	-0.200	1.341	1.774
	A-BC	B	173	GLU	0.08	0.00	0.00	2.84	2.84	-	-	-0.139	0.068

Table 4: Eight atomic types.

Short Name	Atomic names
N_H+	ILE_N__ VAL_N__ LEU_N__ PHE_N__ CYS_N__ MET_N__ ALA_N__ GLY_N__ THR_N__ SER_N__ TRP_N__ TYR_N__ HIS_N__ GLU_N__ GLN_N__ ASP_N__ ASN_N__ LYS_N__ ARG_N__ ARG_NE_ ARG_NH1 ASN_ND2 GLN_NE2 HIS_NE2 TRP_NE1
N+H+	ARG_NH2 LYS_NZ_
O_H0	ILE_O__ VAL_O__ LEU_O__ PHE_O__ CYS_O__ MET_O__ ALA_O__ GLY_O__ THR_O__ SER_O__ TRP_O__ TYR_O__ PRO_O__ HIS_O__ GLU_O__ GLN_O__ ASP_O__ ASN_O__ LYS_O__ ARG_O__ ASP_OD1 GLU_OE1 ASN_OD1 GLN_OE1 PRO_N__ HIS_ND1
O-H0	ASP_OD2 GLU_OE2 ILE_OXT VAL_OXT LEU_OXT PHE_OXT CYS_OXT MET_OXT ALA_OXT GLY_OXT THR_OXT SER_OXT TRP_OXT TYR_OXT PRO_OXT HIS_OXT GLU_OXT GLN_OXT ASP_OXT ASN_OXT LYS_OXT ARG_OXT
O_H+	SER_OG_ THR_OG1 TYR_OH_ HOH_O__
C_NO	ILE_C__ VAL_C__ LEU_C__ PHE_C__ CYS_C__ MET_C__ ALA_C__ GLY_C__ THR_C__ SER_C__ TRP_C__ TYR_C__ PRO_C__ HIS_C__ GLU_C__ GLN_C__ ASP_C__ ASN_C__ LYS_C__ ARG_C__ ILE_CA_ VAL_CA_ LEU_CA_ PHE_CA_ CYS_CA_ MET_CA_ ALA_CA_ GLY_CA_ THR_CA_ SER_CA_ TRP_CA_ TYR_CA_ PRO_CA_ HIS_CA_ GLU_CA_ GLN_CA_ ASP_CA_ ASN_CA_ LYS_CA_ ARG_CA_ ARG_CZ_ ARG_CD_ LYS_CE_ PRO_CD_ ASP_CG_ GLU_CD_ SER_CB_ THR_CB_ ASN_CG_ GLN_CD_
C_NON	MET_SD_ CYS_SG_ LEU_CG_ ILE_CB_ VAL_CB_ LEU_CB_ PHE_CB_ CYS_CB_ MET_CB_ ALA_CB_ TRP_CB_ TYR_CB_ PRO_CB_ HIS_CB_ GLU_CB_ GLN_CB_ ASP_CB_ ASN_CB_ LYS_CB_ ARG_CB_ ARG_CG_ GLU_CG_ GLN_CG_ LYS_CG_ LYS_CD_ PRO_CG_ ILE_CG1 MET_CG_ MET_CE_ ILE_CD1 THR_CG2 ILE_CG2 LEU_CD1 LEU_CD2 VAL_CG1 VAL_CG2
ARC	HIS_CD2 HIS_CE1 HIS_CG_ PHE_CG_ PHE_CD1 PHE_CD2 PHE_CE1 PHE_CE2 PHE_CZ_ TRP_CD1 TRP_CE2 TRP_CH2 TRP_CE3 TRP_CZ3 TRP_CG_ TRP_CD2 TRP_CZ2 TYR_CZ_ TYR_CG_ TYR_CD1 TYR_CD2 TYR_CE1 TYR_CE2

‘H+’: more than one hydrogen
‘H0’: without any hydrogens
‘N/O_’: nitrogen/oxygen atoms without charged
‘N+’: nitrogen atoms with positively charged
‘O-’: oxygen atoms with negatively charged
‘C_NO(N)’: carbon atoms with(out) covalent-bond nitrogen or oxygen
‘ARC’: carbon atoms in an aromatic ring
For ‘XXXYYZZ’ or ‘XXX_YZZ’ or ‘XXX_YZ_’ in the second column, XXX represents a residue type, and _Y or YY denotes an atomic name, while Z or Z_ indicates a specific position of _Y or YY in XXX.

References

1. Fischer TB, Arunachalam KV, Bailey D, Mangual V, Bakhru S, Russo R, Huang D, Paczkowski M, Lalchandani V, Ramachandra C, Ellison B, Galer S, Shapley J, Fuentes E, Tsai J: **The binding interface database (BID): a compilation of amino acid hot spots in protein interfaces.** *Bioinformatics* 2003, **19**(11):1453–1454.

2. Hopf M, Gohring W, Ries A, Timpl R, Hohenester E: **Crystal structure and mutational analysis of a perlecan-binding fragment of nidogen-1**. *Nature structural biology* 2001, **8**(7):634–640.

3. Hubbard SJ, Thornton JM: **'NACCESS', computer program.** Tech. rep., Department of Biochemistry Molecular Biology, University College London 1993.
